# Systematic review of the relationships between sleep duration and health indicators in the early years (0–4 years)

**DOI:** 10.1186/s12889-017-4850-2

**Published:** 2017-11-20

**Authors:** Jean-Philippe Chaput, Casey E. Gray, Veronica J. Poitras, Valerie Carson, Reut Gruber, Catherine S. Birken, Joanna E. MacLean, Salomé Aubert, Margaret Sampson, Mark S. Tremblay

**Affiliations:** 1 Healthy Active Living and Obesity Research Group, Children’s Hospital of Eastern Ontario Research Institute, 401 Smyth Road, Ottawa, ON K1H 8L1 Canada; 2 Faculty of Physical Education and Recreation, University of Alberta, Edmonton, AB T6G 2H9 Canada; 3 Department of Psychiatry, Faculty of Medicine, McGill University, Montreal, QC, H3A 1A1 Canada; 4 Division of Paediatric Medicine, Department of Paediatrics, The Hospital for Sick Children, University of Toronto, Toronto, ON M5G 1X8 Canada; 5 Department of Pediatrics, Faculty of Medicine & Dentistry, University of Alberta, Edmonton, AB T6G 1C9 Canada

**Keywords:** Adiposity, Emotional regulation, Cognitive development, Motor development, Growth, Cardiometabolic health, Physical activity, Sedentary behaviour, Quality of life, Well-being, Injuries, Newborns, Infants, Toddlers, Preschoolers

## Abstract

**Background:**

The objective of this systematic review was to examine for the first time the associations between sleep duration and a broad range of health indicators in children aged 0 to 4 years.

**Methods:**

Electronic databases were searched with no limits on date or study design. Included studies (published in English or French) were peer-reviewed and met the *a priori* determined population (apparently healthy children aged 1 month to 4.99 years), intervention/exposure/comparator (various sleep durations), and outcome criteria (adiposity, emotional regulation, cognitive development, motor development, growth, cardiometabolic health, sedentary behaviour, physical activity, quality of life/well-being, and risks/injuries). The quality of evidence was assessed using the Grading of Recommendations Assessment, Development and Evaluation (GRADE) framework. Due to high levels of heterogeneity across studies, narrative syntheses were employed.

**Results:**

A total of 69 articles/studies (62 unique samples) met inclusion criteria. Data across studies included 148,524 unique participants from 23 countries. The study designs were randomized trials (*n* = 3), non-randomized interventions (*n* = 1), longitudinal studies (*n* = 16), cross-sectional studies (*n* = 42), or longitudinal studies that also reported cross-sectional analyses (*n* = 7). Sleep duration was assessed by parental report in 70% of studies (*n* = 48) and was measured objectively (or both objectively and subjectively) in 30% of studies (*n* = 21). Overall, shorter sleep duration was associated with higher adiposity (20/31 studies), poorer emotional regulation (13/25 studies), impaired growth (2/2 studies), more screen time (5/5 studies), and higher risk of injuries (2/3 studies). The evidence related to cognitive development, motor development, physical activity, and quality of life/well-being was less clear, with no indicator showing consistent associations. No studies examined the association between sleep duration and cardiometabolic biomarkers in children aged 0 to 4 years. The quality of evidence ranged from “very low” to “high” across study designs and health indicators.

**Conclusions:**

Despite important limitations in the available evidence, longer sleep duration was generally associated with better body composition, emotional regulation, and growth in children aged 0 to 4 years. Shorter sleep duration was also associated with longer screen time use and more injuries. Better-quality studies with stronger research designs that can provide information on dose-response relationships are needed to inform contemporary sleep duration recommendations.

**Electronic supplementary material:**

The online version of this article (10.1186/s12889-017-4850-2) contains supplementary material, which is available to authorized users.

## Background

Sleep is essential for healthy cognitive, psychosocial, and physical health [1, 2]. Healthy sleep is generally defined by adequate duration, appropriate timing, good quality, and the absence of sleep disturbances or disorders [3]. Sleep-wake regulation and sleep states evolve rapidly during the first year of life, with continued maturation across childhood [4]. For example, newborns (0–3 months) do not have an established circadian rhythm [5]; this begins to emerge at around 10–12 weeks of age, with sleep becoming more nocturnal between ages 4–12 months [6]. Children continue to take daytime naps between 1 and 4 years of age, and night wakings are common in infancy and early childhood [7]. By age 5, daytime napping typically ceases and overnight sleep duration gradually declines throughout childhood, in part due to a shift to later bedtimes and unchanged wake times [7].

Sleep patterns can vary between individuals and are explained by a complex interplay between genetic, environmental, behavioural, and social factors. For example, factors such as parenting practices and expectations, family routines, cultural preferences, and daycare schedules can all influence sleep [4]. Findings from a recent systematic review of 69,542 infants, toddlers, and preschoolers from 18 countries showed mean reference values and ranges (± 1.96 SD) of 12.8 h/day (9.7–15.9) for infants (< 2 years), and 11.9 h/day (9.9–13.8) for toddlers/preschoolers (ages 2–5 years) [8]. These international normative data can help to determine the normative distribution of sleep duration, but cannot identify duration associated with health benefits.

Although many studies have confirmed the importance of sleep duration for individual health outcomes, to our knowledge no study has attempted to systematically and comprehensively examine the literature on the associations between sleep duration and a broad range of health indicators in children aged 0–4 years. A systematic review can help to determine whether the available evidence supports existing sleep duration recommendations. The National Sleep Foundation recommends that for every 24-h cycle, newborns (0–3 months) obtain 14–17 h of sleep, infants (4–11 months) obtain 12–15 h of sleep, toddlers (1–2 years) obtain 11–14 h of sleep, and preschoolers (3–5 years) obtain 10–13 h of sleep [9]. Similarly, the American Academy of Sleep Medicine recommends that infants (4–11 months) sleep 12–16 h/day, children 1 to 2 years of age sleep 11–14 h/day, and children 3 to 5 years of age sleep 10–13 h/day on a regular basis (including naps) to promote optimal health [10]. Although the ideal amount of sleep may vary from one person to another, sleep duration recommendations are important for surveillance and to inform public policies, interventions, and the general public of healthy sleep behaviours [11, 12].

Therefore, the present work aims to provide a global picture of how sleep duration relates to, or affects, a broad set of health indicators in children aged 0–4 years, and findings from this review will help to better inform sleep duration recommendations for this population and identify future research needs. More specifically, the objective of this systematic review is to examine the relationships between sleep duration and various health indicators in children aged 0–4 years.

## Methods

### Protocol and registration

This review was registered *a priori* with the International Prospective Register of Systematic Reviews (PROSPERO; Registration no. CRD42016040096; available from http://www.crd.york.ac.uk/PROSPERO/display_record.asp?ID=CRD42016040096), and was conducted following the Preferred Reporting Items for Systematic Reviews and Meta-Analyses (PRISMA) statement for reporting systematic reviews and meta-analyses [13].

### Eligibility criteria

The Participants, Interventions, Comparisons, Outcomes, and Study design (PICOS) framework [14] was followed to identify key study concepts in the research question *a priori*, and to facilitate the search process.

### Population

The population included apparently healthy (i.e., general populations, including those with overweight/obesity, but with no diagnosed medical condition) children aged 1 month to 4.99 years for at least one exposure measurement point. Clinical populations (e.g., patients with sleep apnea) were excluded. Subgroups were defined as: newborns (0–3 months), infants (4–11 months), toddlers (1–2 years), and preschoolers (3–4.99 years).

### Intervention (exposure)

The intervention or exposure was sleep duration. Studies were included if they used objective (e.g., polysomnography, actigraphy/accelerometry) or subjective (e.g., proxy-report) measures of sleep duration (or both). This could include actual sleep duration or even time in bed, depending on how it was reported in the studies. Experimental studies were included only if the intervention targeted sleep duration exclusively and not multiple health behaviours (e.g., interventions that targeted both sleep and diet).

### Comparison

Various sleep durations were used for comparison. A comparator or control group was not required for inclusion.

### Outcomes (health indicators)

Ten health indicators were chosen based on the literature, expert input and consensus, and recognition of the importance of including a broad range of health indicators. Five health indicators were identified as *critical* (primary outcomes) by expert agreement: (1) adiposity (e.g., overweight, obesity, body mass index, skinfold thickness, body fat); (2) emotional regulation (e.g., mood, social-emotional problems, stress, hyperactivity/impulsivity); (3) cognitive development (e.g., learning, memory, attention, concentration, language development); (4) motor development (e.g., gross motor skills, fine motor skills, locomotor and object control); and (5) growth. Five health indicators were identified as *important* (secondary outcomes) by expert agreement: (1) cardiometabolic health (e.g., blood pressure, blood lipids, glucose, insulin); (2) sedentary behaviour (e.g., screen time); (3) physical activity (e.g., moderate- to vigorous-intensity physical activity); (4) quality of life/well-being; and (5) risks/injuries.

### Study designs

All study designs, except case studies, were eligible for inclusion in this systematic review. In longitudinal studies, any follow-up length was allowed; however, the exposure had to be assessed at least once during the identified age range. There were no sample size restrictions for studies included in this systematic review. Published peer-reviewed original manuscripts and “in press” articles were eligible for inclusion, as were studies with results posted to a trial registry. Grey literature, book chapters, dissertations and conference abstracts were excluded.

### Information sources and search strategy

A research librarian with expertise in systematic review searching created the electronic search strategy. A second research librarian peer-reviewed it. See Additional file 1: Table S1 for the complete search strategies. The following databases were searched using the Ovid interface, initially in June and again in November 2016: MEDLINE (1946 to November 1, 2016), EMBASE (1980 to 2016 Week 44), PsychINFO (1806 to 2016 October Week 4), and the Cochrane Central Register of Controlled Trials (CENTRAL) (September 2016). Trial registries (https://clinicaltrials.gov and http://who.int/ictrp/en) were searched for registered clinical trials that met our inclusion/exclusion criteria and where results were posted online. Reference lists of relevant reviews were also checked. Studies were included if they were published in English or French.

### Study selection

References were extracted as text files and imported into the Reference Manager Software (Thompson Reuters, San Francisco, CA, USA) for removal of duplicate references. Titles and abstracts of potentially relevant articles were imported to DistillerSR (Evidence Partners, Ottawa, ON, Canada), and were screened independently by two reviewers. Exclusion by both reviewers was needed for a study to be excluded at the first level screening. A full-text copy of each article that met initial screening criteria was obtained, and the same two reviewers independently examined all full-text manuscripts (level 2 screening). Any discrepancies were resolved with a discussion and consensus between the two reviewers. If the reviewers were unable to reach consensus, a third reviewer was asked to examine the article.

### Data extraction

Data extraction was completed in Excel (Microsoft) and checked for accuracy by a second reviewer. Results from the most fully adjusted models were extracted for studies that reported findings from multiple models. Important study features (i.e., author, publication year, study design, country, sample size, age and sex of participants, measure of sleep duration and health outcomes, results, and confounders) were extracted.

### Risk of bias and study quality assessment

A risk of bias assessment was completed for all included studies, as described in the Cochrane Handbook [15]. Briefly, the risk of bias assessment identifies methodological features of each study that can impact confidence in the overall estimate of effect for an outcome. The quality of evidence for each outcome by type of study design was determined using the Grading of Recommendations Assessment, Development and Evaluation (GRADE) framework [16]. The GRADE framework categorizes the quality of evidence into four groups (high, moderate, low, and very low). The quality of evidence rating starts at “high” for randomized studies and at “low” for all other studies (e.g., non-randomized experiments or observational studies). The quality of evidence can be downgraded if there are serious limitations across studies (e.g., serious risk of bias, inconsistency of relative treatment effects, indirectness, imprecision, or other factors) [16]. The quality of evidence assessment was conducted by the lead author (J.-P. Chaput) and verified by the larger review team, including systematic review methodology experts (M. Sampson and A. Jaramillo). Disagreements were resolved by discussion among the team members, if needed.

### Synthesis of results

A meta-analysis was planned in the event that findings were found to be sufficiently homogenous in terms of methodological, statistical, and clinical characteristics. If not sufficiently homogeneous, narrative syntheses were planned.

## Results

### Description of studies

**Fig. 1:**
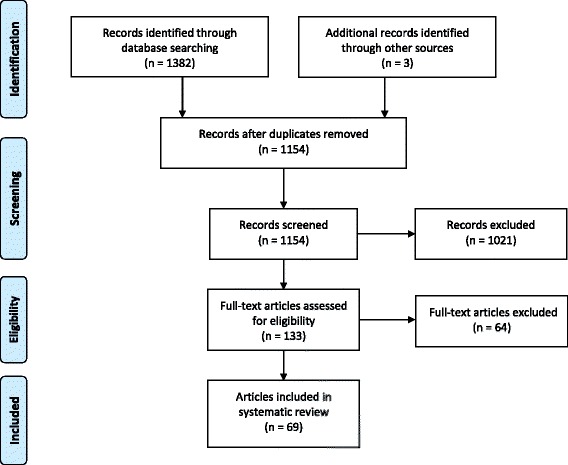
PRISMA flow diagram for the identification, screening, eligibility, and inclusion of studies

As shown in Fig. 1, a total of 1382 records were identified through database searches and an additional three unique records were identified through reference list searches and through the review team and collaborators. Trial registries did not yield any eligible studies. After removing duplicates, a total of 1154 records remained. After titles and abstracts were screened, 133 full-text articles were obtained for further review and 69 articles/studies met the inclusion criteria (from 62 unique samples). Reasons for excluding articles were: not reporting sleep duration as it relates to a health outcome (*n* = 23), no measure of sleep duration (*n* = 15), ineligible age (*n* = 10), participants not apparently healthy (*n* = 8), sleep duration was treated as a covariate or outcome only (*n* = 5), intervention not targeting sleep duration (*n* = 1), not with human participants (*n* = 1), and not original research (i.e., review paper) (*n* = 1). Some studies were excluded for multiple reasons.

Characteristics of studies sorted by outcome indicator are summarized in Additional file 2: Table S2. Data across studies involved 148,524 unique participants. Studies were conducted in 23 different countries from five continents (North America, South America, Europe, Australia/Oceania, and Asia); however, studies were predominantly from North America with White/Caucasian ethnicity. Studies were published between 1992 and 2016, although most were published in the past 5 years. The study designs were randomized trials (*n* = 3), non-randomized interventions (*n* = 1), longitudinal studies (*n* = 16), cross-sectional studies (*n* = 42), or longitudinal studies that also reported cross-sectional analyses (*n* = 7). Sleep duration was measured objectively (polysomnography or actigraphy/accelerometry) in 10 studies, subjectively (parent-report) in 48 studies, and by both actigraphy/accelerometry and a sleep log in 11 studies. It was determined by the review team that a meta-analysis was not possible because of high levels of heterogeneity across studies (see Additional file 2: Table S2), and narrative syntheses were employed instead. All studies are given equal weight in a narrative synthesis of the evidence.

### Data synthesis

#### Adiposity

A total of 31 studies examined the association between sleep duration and adiposity indicators (Table 1 and Additional file 2: Table S2). Among the 13 longitudinal studies, 10 reported that shorter sleep duration was associated with adiposity gain [17–26], 2 reported null findings [27, 28], and 1 reported that longer sleep duration predicted adiposity gain [29]. The quality of evidence remained at “low” for the longitudinal studies. Among the 18 cross-sectional studies, 10 reported a significant association between shorter sleep duration and adiposity [23, 26, 30–37], 7 reported null findings [24, 25, 27, 28, 38–40], and 1 reported that sleep duration was unfavourably associated with adiposity [41]. The quality of evidence remained at “low” for the cross-sectional studies.

**Table 1:** Association between sleep duration and adiposity in children aged 0–4 years

No of studies	Design	Quality Assessment					No of participants	Absolute effect	Quality
		Risk of bias	Inconsistency	Indirectness	Imprecision	Other			
Mean age ranged between 0 and 4.9 years. Data were collected cross-sectionally and up to 9.5 years of follow-up. Sleep duration was assessed by actigraphy or parent report. Adiposity was assessed objectively as body weight, body mass index (absolute, z-score or percentile), waist-for-length ratio, weight status (different definitions for underweight, normal weight, overweight, obese) or % body fat/fat mass/fat mass index (bioelectrical impedance, dual-energy X-ray absorptiometry, skinfolds).									
13	Longitudinal study^a^	No serious risk of bias	No serious inconsistency	No serious indirectness	No serious imprecision	None	31,482	Out of 13 longitudinal analyses, 10 reported a significant association between shorter sleep duration and adiposity gain [17–26], 2 reported null findings [27, 28], and 1 reported that longer sleep duration predicted adiposity gain [29].	LOW
18	Cross-sectional study^b^	No serious risk of bias	No serious inconsistency	No serious indirectness	No serious imprecision	None	30,829	Out of 18 cross-sectional analyses, 10 reported a significant association between shorter sleep duration and adiposity [23, 26, 30–37], 7 reported null findings [24, 25, 27, 28, 38–40], and 1 reported that sleep duration was positively associated with BMI z-scores [41].	LOW

Due to heterogeneity in the measurement of sleep and adiposity, a meta-analysis was not possible

^a^Includes 13 longitudinal studies [17–29]

^b^Includes 18 cross-sectional studies [23–28, 30–41]

#### Emotional regulation

A total of 25 studies examined the association between sleep duration and emotional regulation (Table 2 and Additional file 2: Table S2). The 2 randomized studies (both randomized cross-over trials) showed better self-regulation strategies and emotional responses in the routine sleep versus the sleep restriction condition [42, 43]. The quality of evidence remained at “high” for the randomized trials. There was also 1 non-randomized trial showing a reduced morning cortisol awakening response after sleep restriction [44]. The quality of evidence was downgraded from “low” to “very low” because of a serious risk of imprecision. Among the 5 longitudinal studies, 2 reported that shorter sleep duration was associated with poorer emotional regulation at follow-up [45, 46], while 3 reported null findings [47–49]. The quality of evidence remained at “low” for the longitudinal studies. Among the 17 cross-sectional studies, 8 reported that shorter sleep duration was associated with poorer emotional regulation [50–57], 7 reported null findings [38, 49, 58–62], and 2 reported opposite associations [63, 64]. The quality of evidence was downgraded from “low” to “very low” due to a serious inconsistency in the findings.

**Table 2:** Association between sleep duration and emotional regulation in children aged 0–4 years

No of studies	Design	Quality Assessment					No of participants	Absolute effect	Quality
		Risk of bias	Inconsistency	Indirectness	Imprecision	Other			
Mean age ranged between 1 month and 4.7 years. Intervention studies were between 1 day and 25 days (in-home protocol), and longitudinal studies were up to 6 years. Sleep duration was assessed by actigraphy, polysomnography or parent report. Emotional regulation was assessed through various instruments (e.g. video-recording, cortisol response, or questionnaires).									
2	Randomized trial^a^	No serious risk of bias	No serious inconsistency	No serious indirectness	No serious imprecision	None	22	Nap deprivation resulted in moderate-to-large effects on self-regulation strategies, with decreases in skepticism (d = 0.77; 7% change), negative self-appraisal (d = 0.92; 5% change) and increases in physical self-soothing (d = 0.68; 10% change), focus on the puzzle piece that would not fit (perseveration; d = 0.50; 9% change) and insistence on completing the unsolvable puzzle (d = 0.91; 10% change). After losing daytime sleep, toddlers were less able to engage effectively in a difficult task and reverted to less mature self-regulation strategies than when they were well rested [42]. When sleep restricted, children displayed less confusion in response to neutral pictures, more negativity to neutral and negative pictures, and less positivity to positive pictures. Sleep restriction also resulted in a 34% reduction in positive emotion responses (solvable puzzle), as well as a 31% increase in negative emotion responses and a 39% decrease in confused responses (unsolvable puzzle) [43].	HIGH
1	Non-randomized trial^b^	No serious risk of bias	No serious inconsistency	No serious indirectness	Serious imprecision^c^	None	7	The cortisol awakening response was robust after nighttime sleep, diminished after sleep restriction, and smaller but distinct after morning and afternoon (not evening) naps. Cortisol remained elevated 45 min after morning and afternoon naps [44].	VERY LOW
5	Longitudinal study^d^	No serious risk of bias	No serious inconsistency	No serious indirectness	No serious imprecision	None	46,959	Out of 5 longitudinal analyses, 2 reported that shorter sleep duration was associated with poorer emotional regulation at follow-up [45, 46] while 3 reported null findings [47–49].	LOW
17	Cross-sectional study^e^	No serious risk of bias	Serious inconsistency^f^	No serious indirectness	No serious imprecision	None	16,536	Out of 17 cross-sectional analyses, 8 reported that shorter sleep duration was associated with poorer emotional regulation [50–57], 7 reported null findings [38, 49, 58–62], and 2 reported opposite associations [63, 64].	VERY LOW

Due to heterogeneity in the measurement of sleep and emotional regulation, a meta-analysis was not possible

^a^Includes 2 randomized cross-over studies [42, 43]

^b^Includes 1 non-randomized intervention [44]

^c^Only one study was published with a sample size of *N* = 7 so the risk of imprecision is high (the quality of evidence was downgraded from “low” to “very low”)

^d^Includes 5 longitudinal studies [45–49]

^e^Includes 17 cross-sectional studies [38, 49–64]

^f^Studies reported mixed findings (the quality of evidence was downgraded from “low” to “very low”)

#### Cognitive development

A total of 16 studies examined the association between sleep duration and cognitive development (Table 3 and Additional file 2: Table S2). One randomized trial examined this association [65] and found that the number of correct answers in an explicit recognition task was significantly higher in the nap condition compared to the wake (sleep restriction) condition; however, implicit memory (priming task) did not differ between conditions. The quality of evidence remained at “high” for this randomized trial. The 4 longitudinal studies that examined the relationships between sleep duration and cognitive development provided mixed findings, although they had mainly favourable associations or null findings [66–69]. The quality of evidence for longitudinal studies remained at “low”. Finally, of 11 cross-sectional studies, 7 reported null findings [38, 51, 55, 70–73], 3 reported that shorter sleep duration was associated with poorer cognitive function [57, 74, 75], and 1 reported opposite associations [76]. The quality of evidence remained at “low” for the cross-sectional studies.

**Table 3:** Association between sleep duration and cognitive development in children aged 0–4 years

No of studies	Design	Quality Assessment					No of participants	Absolute effect	Quality
		Risk of bias	Inconsistency	Indirectness	Imprecision	Other			
Mean age ranged between 6 months and 4.9 years. Data were collected cross-sectionally and up to 3 years of follow-up. Sleep duration was assessed by actigraphy or parent report. Cognition was measured by various instruments including memory tasks, imitation tasks, neuropsychological tests, interviews, scales of intelligence or questionnaires.									
1	Randomized trial^a^	No serious risk of bias	No serious inconsistency	No serious indirectness	No serious imprecision	None	23	The number of correct answers in an explicit recognition task was significantly higher in the nap (control) compared to the wake (sleep-restricted) condition, whereas implicit memory (priming task) did not differ between conditions [65].	HIGH
4	Longitudinal study^b^	No serious risk of bias	No serious inconsistency	No serious indirectness	No serious imprecision	None	438	Children getting higher proportions of their sleep at night as infants (i.e. 1 year) were found to perform better on executive functions, but did not show better general cognition [66]. Higher proportions of total sleep occurring at night time, at both 12 and 18 months, were associated with better performance on executive tasks, especially those involving a strong impulse control component. However, the total sleep duration at 12 and 18 months was not associated with executive functioning at 18 and 26 months. Sleep duration at 12 months was not correlated with 18 month working memory (*r* = −0.11, *p* > 0.05), 26 month conflict executive functioning (*r* = −0.10, *p* > 0.05) or 26 month impulse control (*r* = −0.06, *p* > 0.05). Sleep duration at 18 months was not correlated with 18 month working memory (*r* = −0.16, *p* > 0.05), 26 month conflict executive functioning (*r* = 0.09, *p* > 0.05) or 26 month impulse control (r = −0.16, *p* > 0.05) [67]. The number of daytimenaps was positively associated with both predicted expressive (*p* = 0.062) and receptive vocabulary growth (*p* = 0.006), whereas the length of nighttime sleep was negatively associated with rate of predicted expressive vocabulary growth (*p* = 0.045) [68]. Children who had 8 h or more of sleep had significantly higher General Conceptual Ability (GCA) scores than those with 7 h or less of sleep by 35.53 points at age 3. Children with more than 10 h of sleep had higher GCA scores at age 3 compared to children with 8–9 h or less of sleep (233.91 vs. 203.92, respectively) [69].	LOW
11	Cross-sectional study^c^	No serious risk of bias	No serious inconsistency	No serious indirectness	No serious imprecision	None	10,838	Out of 11 cross-sectional analyses, 7 reported null findings [38, 51, 55, 70–73], 3 reported that shorter sleep duration was associated with poorer cognitive function [57, 74, 75], and 1 reported opposite associations [76].	LOW

Due to heterogeneity in the measurement of sleep and cognition, a meta-analysis was not possible

^a^Randomized cross-over study [65]

^b^Includes 4 longitudinal studies [66–69]

^c^Includes 11 cross-sectional studies [38, 51, 55, 57, 70–76]

#### Motor development

Two cross-sectional studies examined the association between sleep duration and motor development (Table 4 and Additional file 2: Table S2). Both studies reported no associations between sleep duration, and gross and fine motor skills [38, 51]. The quality of evidence remained at “low” for the cross-sectional studies.

**Table 4:** Association between sleep duration and motor development in children aged 0–4 years

No of studies	Design	Quality Assessment					No of participants	Absolute effect	Quality
		Risk of bias	Inconsistency	Indirectness	Imprecision	Other			
Mean age ranged between 7.4 months and 13 months. Data were collected cross-sectionally only. Sleep duration was assessed by actigraphy or parent report. Motor development was assessed using the Ages and Stages Questionnaire in both studies.									
2	Cross-sectional study^a^	No serious risk of bias	No serious inconsistency	No serious indirectness	No serious imprecision	None	1403	Sleep duration was not associated with gross and fine motor skills [38, 51].	LOW

Due to the fact that only two studies were published on sleep duration and motor development (with different methodologies and age groups), a meta-analysis was not possible

^a^Includes 2 cross-sectional studies [38, 51]

#### Growth

Two studies examined the relationship between sleep duration and linear growth (Table 5 and Additional file 2: Table S2). The longitudinal study by Lampl et al. [29] showed that higher total daily sleep hours and number of sleep bouts were significantly associated with growth in infant length. The quality of evidence was downgraded from “low” to “very low” for this study because of a serious risk of bias. In the cross-sectional study [77], sleep was assessed both objectively and subjectively in 6-month-old infants. The authors reported that shorter actigraphy-measured sleep duration was associated with higher weight-for-length ratio in girls only. The results also showed that, in the total sample, shorter night sleep duration (as reported by parents) was associated with higher weight-for-length ratio and weight above the expected weight for length. The quality of evidence was downgraded from “low” to “very low” due to a serious risk of imprecision.

**Table 5:** Association between sleep duration and growth in children aged 0–4 years

No of studies	Design	Quality Assessment					No of participants	Absolute effect	Quality
		Risk of bias	Inconsistency	Indirectness	Imprecision	Other			
Mean age ranged between 4 months and 17 months. Data were collected cross-sectionally and up to 13 months. Sleep duration was assessed by actigraphy or parent report. Growth was assessed using the maximum stretch technique and using weight above the expected weight for length.									
1	Longitudinal study^a^	Serious risk of bias^b^	No serious inconsistency	No serious indirectness	No serious imprecision	None	23	Saltatory length growth was associated with increased total daily sleep hours (*p* < 0.001) and number of sleep bouts (*p* = 0.001). Subject-specific probabilities of a growth saltation associated with sleep included a mean odds ratio of 1.20 for each additional hour of sleep (*n* = 8, 95% CI 1.15–1.29) and 1.43 for each additional sleep bout (*n* = 12, 95% CI 1.21–2.03) [29].	VERY LOW
1	Cross-sectional study^c^	No serious risk of bias	No serious inconsistency	No serious indirectness	Serious imprecision^d^	None	139,305	Using actigraphy, sleep duration was associated with weight-to-length ratio (*r* = −0.47, *p* < 0.01) in girls only. Using the questionnaire, night sleep duration was associated with weight-to-length ratio (*r* = −0.26, *p* < 0.05) and weight above the expected weight for length (*r* = −0.25, *p* < 0.05) in the total sample [77].	VERY LOW

^a^Includes 1 longitudinal study [29]

^b^Sleep duration was parent-reported with no psychometric properties reported. Therefore, the quality of evidence was downgraded from “low” to “very low”

^c^Includes 1 cross-sectional study [77]

^d^Only one study was published, including a convenience sample of infants and showing differences between boys and girls with the use of actigraphy, so the risk of imprecision is high. Therefore, the quality of evidence was downgraded from “low” to “very low”. Due to the fact that only two studies were published on sleep duration and growth, a meta-analysis was not possible

#### Cardiometabolic health

No studies examined the association between sleep duration and cardiometabolic biomarkers in children aged 0–4 years.

#### Sedentary behaviour

A total of 5 studies (1 longitudinal study and 4 cross-sectional studies) examined the association between sleep duration and screen time (Table 6 and Additional file 2: Table S2). The longitudinal study showed that longer sleep duration at 4 years of age was associated with less television viewing and computer use at 6 years of age [22]. The quality of evidence was downgraded from “low” to “very low” due to a serious risk of bias. The 4 cross-sectional studies [31, 78–80] showed that shorter sleep duration was associated with more screen time. The quality of evidence was downgraded from “low” to “very low” because of a serious risk of bias.

**Table 6:** Association between sleep duration and sedentary behaviour in children aged 0–4 years

No of studies	Design	Quality Assessment					No of participants	Absolute effect	Quality
		Risk of bias	Inconsistency	Indirectness	Imprecision	Other			
Mean age ranged between 6 months and 4.5 years. Data were collected cross-sectionally and up to 4 years. Sleep duration was assessed by parent report. Sedentary behaviors (screen time) were assessed using time-use diaries or questionnaires.									
1	Longitudinal study^a^	Serious risk of bias^b^	No serious inconsistency	No serious indirectness	No serious imprecision	None	2984	Sleep duration at 4 years of age was inversely associated with television viewing (β = −0.07, *p* = 0.003) and computer use (β = −0.04, *p* = 0.001) at 6 years of age [22].	VERY LOW
4	Cross-sectional study^c^	Serious risk of bias^d^	No serious inconsistency	No serious indirectness	No serious imprecision	None	42,751	Short sleep duration was associated with time spent watching TV (OR: 1.65, 95% CI 1.23–2.21 per additional hour/24 h) in boys. In girls, the association was not significant (*p* = 0.75) [31]. Infants who were exposed to screen media in the evening at 12 months of age had a 28-min lower nighttime sleep duration on weekdays. Moreover, infants who were exposed to screen media in the evening at age 6 months and 12 months had shorter 12-month nighttime sleep duration compared with those who were not exposed to screen media after 7 pm at both ages [78]. Watching more than an hour of TV in the evening was associated with short sleep duration (OR = 1.89, 95% CI 1.26–2.84). However, the association was not significant with watching more than an hour of TV in the morning (OR = 1.13, 95% CI 0.80–1.58) [79]. Short sleep duration was associated with longer hours spent watching television (OR = 1.91, 95% CI 1.26–2.90 for ≥4 h/day) and playing computer games (OR = 1.62, 95% CI 1.18–2.23 for ≥2 h/day) compared to not watching/playing [80].	VERY LOW

Due to heterogeneity in the measurement of sleep and sedentary behaviors, a meta-analysis was not possible

^a^Includes 1 longitudinal study [22]

^b^Sleep duration was parent-reported with no psychometric properties reported. Therefore, the quality of evidence was downgraded from “low” to “very low”

^c^Includes 4 cross-sectional studies [31, 78–80]

^d^Sleep duration was parent-reported with no psychometric properties reported. Therefore, the quality of evidence was downgraded from “low” to “very low”

#### Physical activity

Four studies (1 longitudinal study and 3 cross-sectional studies) examined the association between sleep duration and physical activity (Table 7 and Additional file 2: Table S2). The longitudinal study showed that sleep duration at 4 years of age was not associated with level of physical activity at 6 years of age [22]. The quality of evidence was downgraded from “low” to “very low” due to a serious risk of bias. The 3 cross-sectional studies [30, 31, 81] showed either favourable (i.e., longer sleep duration was associated with more physical activity) or null findings. The quality of evidence remained at “low” for the cross-sectional studies.

**Table 7:** Association between sleep duration and physical activity in children aged 0–4 years

No of studies	Design	Quality Assessment					No of participants	Absolute effect	Quality
		Risk of bias	Inconsistency	Indirectness	Imprecision	Other			
Mean age ranged between 20 months and 4.5 years. Data were collected cross-sectionally and up to 4 years. Sleep duration was assessed by parent report. Physical activity was assessed using accelerometers, time-use diaries or questionnaires.									
1	Longitudinal study^a^	Serious risk of bias^b^	No serious inconsistency	No serious indirectness	No serious imprecision	None	2984	Sleep duration at 4 years of age was not associated with physical activity at 6 years of age (β = −0.02, 95% CI −0.09-0.03) [22].	VERY LOW
3	Cross-sectional study^c^	No serious risk of bias	No serious inconsistency	No serious indirectness	No serious imprecision	None	2272	Longer nighttime sleep duration was associated with more physical activity (MVPA min/day: *r* = 0.19, *p* = 0.012; activity counts: *r* = 0.21, *p* = 0.006). In multivariable models, nighttime sleep duration was positively associated with physical activity (β = 0.332, *p* = 0.017) [30]. Sleep duration was not associated with physical activity in either boys (*p* = 0.89) or girls (*p* = 0.41) [31]. Total daily sleep duration was positively associated with physical activity in boys only (OR = 1.04, 95% CI 1.02–1.07) [81].	LOW

Due to heterogeneity in the measurement of sleep and physical activity, a meta-analysis was not possible

^a^Includes 1 longitudinal study [22]

^b^Sleep duration was parent-reported with no psychometric properties reported. Therefore, the quality of evidence was downgraded from “low” to “very low”

^c^Includes 3 cross-sectional studies [30, 31, 81]

#### Quality of life/well-being

Only 1 study examined the association between sleep duration and quality of life/well-being (Table 8 and Additional file 2: Table S2). This longitudinal study found that short sleep duration at 3 years of age (< 10 h versus > 11 h) was not associated with poor quality of life at ~13 years of age [82]. The quality of evidence was downgraded from “low” to “very low” because of a serious risk of bias.

**Table 8:** Association between sleep duration and quality of life/well-being in children aged 0–4 years

No of studies	Design	Quality Assessment					No of participants	Absolute effect	Quality
		Risk of bias	Inconsistency	Indirectness	Imprecision	Other			
Children were 3 years of age and followed until first-year junior high school (approximately 13 years old). Data were collected longitudinally (approximately a 10-year follow-up period). Sleep duration was assessed by parent report. Quality of life was assessed using the Dartmouth Primary Care Cooperative Project (COOP) charts.									
1	Longitudinal study^a^	Serious risk of bias^b^	No serious inconsistency	No serious indirectness	No serious imprecision	None	9674	Short sleep duration at 3 years of age (< 10 h vs. > 11 h) was not associated with quality of life at age ~13 years (OR = 1.15, 95% CI 0.99–1.33, *p* = 0.06) [82].	VERY LOW

Due to the fact that only one study was published on sleep duration and quality of life/well-being, a meta-analysis was not possible

^a^Includes 1 longitudinal study [82]

^b^Sleep duration was parent-reported with no psychometric properties reported. Therefore, the quality of evidence was downgraded from “low” to “very low”

#### Risks/injuries

Three cross-sectional studies examined the association between sleep duration and risks/injuries in children aged 0–4 years (Table 9 and Additional file 2: Table S2). Koulouglioti et al. [83] reported that children with shorter sleep duration sustained a higher number of medically attended injuries. Likewise, Boto et al. [84] reported that a sleep duration shorter than 8 h per day was associated with an increased risk of accidental falls. In contrast, Owens et al. [85] did not find an association between sleep duration and injury risk. The quality of evidence remained at “low” for the cross-sectional studies.

**Table 9:** Association between sleep duration and risks/injuries in children aged 0–4 years

No of studies	Design	Quality Assessment					No of participants	Absolute effect	Quality
		Risk of bias	Inconsistency	Indirectness	Imprecision	Other			
Mean age ranged between 18 months and 4.9 years. Data were collected cross-sectionally only. Sleep duration was assessed by parent report. Risks/injuries were assessed using medical record data, the Injury Behavior Checklist, interviews, or chart reviews of injuries.									
3	Cross-sectional study^a^	No serious risk of bias	No serious inconsistency	No serious indirectness	No serious imprecision	None	2382	Children with shorter sleep duration sustained a higher number of medically attended injuries (b = 0.1759, *p* < 0.05) [83]. Usual sleep duration shorter than 8 h was associated with an increased risk of accidental falls (OR = 2.7, 95% CI 1.2–6.1) [84]. The Children’s Sleep Habits Questionnaire (CSHQ) sleep duration score did not significantly differ between the high injury and low injury groups (5.93 ± 1.03 vs. 6.36 ± 0.96, respectively, *p* = 0.09). Also, the CSHQ sleep duration score did not significantly differ between the high-injury-behavior and the low-injury-behavior groups (5.73 ± 2.10 vs. 4.32 ± 1.92, respectively, p not provided) after Bonferroni correction. The Pearson correlation coefficient between sleep duration and the total Injury Behavior Checklist score was *r* = 0.32, *p* = 0.005. To specifically examine the relationship between parent-reported sleep duration and injuries and injury behavior, they divided the group by median split for sleep duration (low sleep < 690 min, high sleep ≥690 min). There were no significant differences in the number of injuries in the past 2 years or in the Injury Behavior Checklist total score [85].	LOW

^a^Includes 3 cross-sectional studies [83–85]. Due to heterogeneity in the measurement of sleep and risks/injuries, a meta-analysis was not possible

#### Summary of findings

A high-level summary of findings by health outcome can be found in Table 10. Overall, studies tended to show favourable associations between sleep duration and adiposity (20/31 studies), emotional regulation (13/25 studies), growth (2/2 studies), screen time (5/5 studies), and risks/injuries (2/3 studies). However, no association was found between sleep duration and motor development (only 2 studies) and quality of life (only 1 study), and the evidence was mixed for cognitive development and physical activity indicators. It is difficult to establish the optimal amount of sleep associated with favourable health outcomes based on the available evidence. Most of the evidence was correlational in nature or compared groups with different cut-points for short and long sleep duration. However, longer sleep durations, when compared to shorter sleep durations, were generally associated with better outcomes in the studies synthesized herein, and the pattern of associations did not differ by the age group examined (i.e., infants, toddlers, and preschoolers).

**Table 10:** High-level summary of findings by health indicator

Health Indicator	# of studies	Quality of Evidence	Summary of Findings
Critical			
Adiposity	31	Low	*N* = 20 studies reported a significant association between shorter sleep duration and adiposity. *N* = 9 studies reported null findings. *N* = 2 studies reported a significant association between longer sleep duration and adiposity.
Emotional Regulation	25	Very Low to High	*N* = 13 studies reported a significant association between shorter sleep duration and poorer emotional regulation. *N* = 10 studies reported null findings. *N* = 2 studies reported a significant association between longer sleep duration and poorer emotional regulation.
Cognitive Development	16	Low to High	*N* = 6 studies reported a significant association between shorter sleep duration and poorer cognitive function. *N* = 8 reported null findings. *N* = 2 study reported a significant association between longer sleep duration and poorer cognitive function.
Motor Development	2	Low	*N* = 2 studies reported null findings.
Growth	2	Very Low	*N* = 2 studies reported better growth with longer sleep duration.
Important			
Sedentary Behavior	5	Very Low	*N* = 5 studies reported shorter sleep duration was associated with more screen time.
Physical Activity	4	Low to Very Low	*N* = 2 studies reported longer sleep duration was associated with more physical activity. *N* = 2 studies reported null findings.
Risks/Injuries	3	Low	*N* = 2 studies reported a higher risk of injuries with shorter sleep duration. *N* = 1 study reported null findings.
Quality of Life/Well-Being	1	Very Low	*N* = 1 study reported null findings.
Cardio-Metabolic Health	0	N/A	N/A

The number of studies is more than *N* = 69 because some papers had more than one outcome measure and/or study design

## Discussion

This systematic review synthesized peer-reviewed scientific evidence from 69 articles/studies examining the relationships between sleep duration and key health indicators in children aged 0–4 years. The overall quality of evidence ranged from “very low” to “high” across study designs and health indicators. Collectively, shorter sleep duration was generally associated with higher adiposity, poorer emotional regulation, impaired growth, more screen time, and higher risk of injuries. However, the evidence was mixed for cognitive development and physical activity, and null findings were reported for motor development and quality of life. Also, no studies examined the association between sleep duration and cardiometabolic biomarkers in this population. Overall, this comprehensive assessment of available evidence should encourage efforts aimed at promoting the importance of sleep duration for overall health in children aged 0–4 years.

Adiposity (*n* = 31 studies) and emotional regulation (*n* = 25 studies) were the health indicators with the highest number of studies in the present systematic review. This is in agreement with our previous systematic review examining the associations between sleep duration and health indicators in school-aged children and youth [2]. However, the findings from these two health indicators in the current paper are more mixed than those found in the children and youth review. Potential reasons to explain this difference include: (1) differences in measurement tools used to assess sleep duration and health outcomes; (2) differences in confounding factors; (3) differences in development stages; (4) differences in the robustness of study designs; and (5) the likelihood that it is more difficult to find associations with adverse health indicators in a younger and healthier population of children, as the outcomes explored in this review are likely to manifest over time if short sleep duration is prolonged .

Many tools have been used to assess emotional regulation in the studies reviewed herein. These included video-recording, various questionnaires, and even cortisol response. It is debatable whether cortisol awakening response (CAR) is an emotional regulation indicator, but it fit our inclusion/exclusion criteria as a stress marker. The non-randomized intervention that examined CAR after sleep restriction [44] showed that CAR was robust after nighttime sleep, diminished after sleep restriction, and was smaller but still distinct after morning and afternoon naps. Although the authors indicated in their article that reduced CAR after shortened sleep suggests a decreased ability to deal with environmental stressors, this viewpoint is not unanimous in the scientific community.

Findings of studies included in the present systematic review are consistent with current sleep duration recommendations, and we are not advocating that they should be changed. However, based on the results of our review—which gathered the best evidence available in this field of research—it is clear that, currently, the evidence being used to inform sleep duration recommendations in the early years is weak, suggesting that expert opinion is needed until more and better research is conducted. There is an urgent need for higher-quality studies that can help to better inform recommendations for sleep duration in this population. For example, the available evidence relies heavily on cross-sectional studies that use parent-reported sleep durations. Multiple age groups were also grouped together, despite obvious differences in development. Most importantly, the current evidence is largely correlational in nature, and there is a clear need for dose-response curves with multiple time points of sleep duration that can provide a better idea of optimal sleep duration ranges. In an experimental context, this means examining how health indicators change in response to sleep restriction/extension interventions. In observational studies, this means comparing several categories of sleep duration in relation to health indicators rather than using continuous data in order to have a better sense of dose-response gradient. Ideally, results would be reported for narrower age groups that are aligned with the current sleep duration recommendations (i.e., newborns [0–3 months], infants [4–11 months], toddlers [1–2 years], preschoolers [3–5 years]); development progresses rapidly in the early years and many factors can confound the associations (e.g., growth, eating habits, environment, locomotion).

The National Sleep Foundation in the USA recommends that in each 24-h cycle, newborns (0–3 months) obtain 14–17 h of sleep, infants (4–11 months) obtain 12–15 h of sleep, toddlers (1–2 years) obtain 11–14 h of sleep, and preschoolers (3–5 years) obtain 10–13 h of sleep [9]. The American Academy of Sleep Medicine issued similar recommendations in 2016 [10]. To develop their guidelines, both organizations relied on a multidisciplinary expert panel to evaluate the latest scientific evidence, including a consensus and voting process. The present systematic review will also help to inform the *Canadian 24-Hour Movement Guidelines for the Early Years (0–4 Years): An Integration of Physical Activity, Sedentary Behaviour, and Sleep* [86].

Existing sleep duration recommendations provide ranges and imply that a U-shaped association exists between sleep duration and health outcomes, with one side of the “U” representing short sleep duration and the other side representing long sleep duration. It is increasingly recognized that the two sides of this U-shape have different health impacts [87]. While insufficient sleep is a stressor for the metabolism and is consistently associated with adverse health outcomes, excessively long sleep duration may interfere with children’s exploration of their physical and social environment and thereby possibly impede their optimal development [88]. Thus, it is logical for public health sleep duration guidelines to recommend a range to represent “healthy” or “optimal” sleep durations rather than a threshold value.

The present systematic review focused on sleep duration only. However, many other important factors beyond sleep quantity should be considered in the development of sleep recommendations, including aspects of sleep quality such as sleep efficiency (i.e., proportion of the sleep opportunity actually spent in sleep), timing (i.e., bedtime/wake-up times and naps), sleep architecture (i.e., sleep stages), consistency (i.e., day-to-day variability, seasonal changes), and sleep consolidation (i.e., organization of sleep across the night). The National Sleep Foundation recently published evidence-based recommendations and guidance to the public regarding indicators of good sleep quality across the lifespan [89]. Overall, the panel members agreed that most sleep continuity variables (e.g., sleep latency, number of awakenings >5 min, wake after sleep onset, and sleep efficiency) were appropriate indicators of good sleep quality. However, there was less or no consensus regarding sleep architecture or nap-related variables as indicators of good sleep quality.

Sleep duration in the early years is generally comprised of both daytime and nighttime sleep. However, it has been reported that daytime sleep and nighttime sleep may not have the same effects on health, with positive effects of sleep duration suggested to relate to the stage in sleep transition from polyphasic to monophasic sleep (the stage at which naps cease) [90]. The same systematic review also concluded that beyond the age of 2 years, napping is associated with later sleep onset at night as well as reductions in both sleep quality and duration, suggesting that clinicians should investigate napping patterns in children who present with sleep problems [90]. As discussed extensively in previous papers, many healthy sleep practices can help to achieve age-appropriate amounts of sleep, including having a consistent bedtime routine and removing screens from children’s bedrooms [88, 91].

It is also well-known that parent-reported sleep duration overestimates actual sleep duration compared with objective measures [92]. This can have implications for sleep duration recommendations if future studies rely more heavily on objective assessments of sleep, because this will require extrapolation of objectively measured sleep durations to real-world conditions. Most of the studies synthesized in the present work utilized subjective measures of sleep with no psychometric properties, and results may have more ecological validity.

A number of limitations and research gaps have been highlighted in the discussion section already. However, other limitations of the present systematic review should be mentioned. First, the high level of heterogeneity across studies precluded conducting meta-analyses, and all studies were weighted equally. Second, the present systematic review included only articles published in English or French, meaning any relevant studies published in other languages were excluded. Third, the risk of publication bias (i.e., an over-representation of studies with significant findings) is always a possibility in science. Finally, many studies did not adjust for important confounding factors (e.g., diet when examining adiposity as an outcome measure), thereby impacting our confidence in the estimates of effect for the outcome measures.

## Conclusions

The present article was the first to systematically examine the associations between sleep duration and a broad range of key health indicators in children aged 0–4 years. We provide support for previous evidence showing that shorter sleep duration is associated with adverse health indicators in some areas of physical and mental health; however, the synthesized evidence relies heavily on cross-sectional study designs and parent-reported sleep durations, and combines multiple ages together despite clear differences in development. To better inform sleep recommendations, scientists should conduct and publish higher-quality studies in this population to have a better idea of dose-response relationships. Robust sleep guidelines should be based on the best available evidence, expert consensus, stakeholder consultation, and consideration of values and preferences, applicability, feasibility, resource use, and equity [86]. There is a clear need for more and better studies in this young age group, which is an important time for growth and development.

## Additional files

Additional file 1: Table S1. Search strategy for the systematic review. (DOC 175 kb)

Additional file 2: Table S2. Summary of studies included in the systematic review sorted by (whenever possible) outcome indicator, study design, age group, and sleep assessment (objective, then subjective). (DOC 254 kb)

## Abbreviations

CAR: Cortisol awakening response
CENTRAL: Cochrane Central Register of Controlled Trials
GRADE: Grading of Recommendations Assessment, Development and Evaluation
PICOS: Participants, interventions, comparisons, outcomes, and study design
PRISMA: Preferred Reporting Items for Systematic Reviews and Meta-Analyses
PROSPERO: International Prospective Register of Systematic Reviews
