# Supplementary material for: Systematic review of the relationships between sleep duration and health indicators in the early years (0–4 years)
Source: BMC Public Health. 2017 Nov 20;17(Suppl 5):855. doi: 10.1186/s12889-017-4850-2 (PMC5773910; doi:10.1186/s12889-017-4850-2)
Supplement: Supplementary file 1 — Search strategy for the systematic review. (DOC 175 kb) [file 12889_2017_4850_MOESM1_ESM.doc]

**Additional File 1: Table S1.** Search strategy for the systematic review.

**MEDLINE**

| 1. Sleep/ |
| --- |
| 2. (sleep adj3 duration).tw. |
| 3. exp *Sleep Apnea Syndromes/ and (apnea or apnoea).ti. |
| 4. (1 or 2) not 3 |
| 5. Polysomnography/ |
| 6. Accelerometer/ or Accelerometry/ |
| 7. Actigraphy/ |
| 8. (polysomnogr* or actigr* or accelerom*).tw. |
| 9. ((objectiv* adj3 measur*) or (direct* adj3 measure*)).tw. |
| 10. (Sleep* adj3 (report or questionnaire* or index or eval* or diary or diaries or log* or journal*)).tw. |
| 11. (self report* or proxy report*).tw. |
| 12. or/5-11 |
| 13. 4 and 12 |
| 14. limit 13 to ("infant (1 to 23 months)" or "preschool child (2 to 5 years)") |
| 15. 13 and (pre-school* or preschool* or early childhood).tw,kf. |
| 16. 14 or 15 |
| 17. limit 16 to (journal article or published erratum or "retraction of publication") |
| 18. limit 17 to (english or french) |
| 19. Sedentary Lifestyle/ |
| 20. (sedentary or inactiv* or (lack adj2 activity)).tw,kw. |
| 21. ((low adj3 energy expend*) or physical* inactiv*).tw,kw. |
| 22. ((chair or stroller or car or automobile* or auto or motor vehicle* or bus or indoor* or in-door or computer) adj3 time).tw,kw. |
| 23. sitting.tw,kw. |
| 24. Television/ or Video Games/ or Software/ or Videodisc Recording/ or Cartoons as Topic/ or Motion Pictures as Topic/ |
| 25. exp Internet/ or exp Computers, handheld/ |
| 26. Communications Media/ or Mass Media/ |
| 27. (television or screentime or ((screen or computer) adj3 time) or ((watch* or view*) adj2 (dvd* or video*)) or screen media or social media or video gam* or videogam* or computer gam* or electronic gam*).tw,kw. |
| 28. (Smartphone* or ipad or apps or app or mobile applications).tw,kw. |
| 29. screen based entertainment.tw,kw. |
| 30. Physical Activity.mp. |
| 31. exp Exercise/ |
| 32. exp Exercise Movement Techniques/ |
| 33. exp Exercise Therapy/ |
| 34. Physical Exertion/ |
| 35. exp "Physical Education and Training"/ |
| 36. exp Sports/ |
| 37. (sport$ or bicycl$ or swim$ or walk$ or run$ or jog$).tw,kf. |
| 38. (physical$ adj2 activ$).tw,kf. |
| 39. (aerobic adj2 (train$ or active$)).tw,kf. |
| 40. "Play and Playthings"/ and (activ* or outdoor*).tw,kf. |
| 41. ((activ* or outdoor*) adj3 play*).tw,kf. |
| 42. playground*.tw,kf. |
| 43. active.ti. and (space* or behavio?r* or transport* or commut* or neighbo?rhood* or park* or game* or gaming or lifestyle).mp. |
| 44. (active adj3 (space* or behavio?r* or transport* or commut* or neighbo?rhood* or park* or game* or gaming or lifestyle)).tw,kf. |
| 45. prone position*.mp. or floor time.tw,kf. |
| 46. ((abdomen or stomach or tummy or belly) adj2 (time or play)).tw,kf. |
| 47. exp obesity/ |
| 48. obes*.tw,kf. |
| 49. exp overweight/ |
| 50. (overweight or over-weight).tw,kf. |
| 51. exp Body Fat Distribution/ |
| 52. exp body composition/ |
| 53. (adipos* or fat).mp. |
| 54. waist.mp. |
| 55. Skinfold Thickness/ |
| 56. (skin fold* or skinfold*).tw,kf. |
| 57. (body composition* or BMI or body mass index).tw,kf. |
| 58. exp "body weights and measures"/ |
| 59. (bio-impedance analysis or BIA).tw,kf. |
| 60. Absorptiometry, Photon/ |
| 61. (absorptiomet* or densitometr* or photodensitometr* or DXA or DEXA).tw,kf. |
| 62. Physical Fitness/ |
| 63. (physical conditioning or fitness).tw,kf. |
| 64. Range of Motion, Articular/ |
| 65. Postural Balance/ |
| 66. Metabolic Syndrome X/ |
| 67. Insulin Resistance/ |
| 68. ((metabolic adj2 syndrome) or syndrome x).tw,kf. and Risk Factors/ |
| 69. exp blood glucose/ or exp diabetes mellitus, type 2/ |
| 70. Hemoglobin A, Glycosylated/ |
| 71. HbA1c.tw,kf. |
| 72. exp Cardiovascular Diseases/ |
| 73. ((cardiovascular or heart or vascular) adj2 risk$).tw,kf. |
| 74. exp Hypertension/ |
| 75. (hypertens* or high blood pressure).tw,kf. |
| 76. exp blood pressure determination/ |
| 77. blood pressure/ |
| 78. Hypercholesterolemia/ |
| 79. exp Hyperlipidemias/ |
| 80. ((cardiovascular or heart or vascular) adj2 risk$).tw,kf. |
| 81. exp Hypertension/ |
| 82. (hypertens* or high blood pressure).tw,kf. |
| 83. exp blood pressure determination/ |
| 84. blood pressure/ |
| 85. Hypercholesterolemia/ |
| 86. exp Hyperlipidemias/ |
| 87. (cholesterol or hypercholester* or hyperlipid* or dyslipid*).tw,kf. |
| 88. exp Psychomotor Performance/ |
| 89. motor development.tw,kf. |
| 90. Motor Activity/ |
| 91. Gross motor skill*.tw,kf. |
| 92. (fine motor skill* or locomotor control or object control).tw,kf. |
| 93. exp "Wounds and Injuries"/ |
| 94. exp Child Development Disorders/ |
| 95. Child Development/ |
| 96. Developmental milestone*.mp. |
| 97. gd.fs. |
| 98. Child Behavior Disorders/ |
| 99. Child Behavior/ |
| 100. exp Adaptation, Psychological/ |
| 101. (pro-social behav* or prosocial behav* or social behav*).tw,kf. |
| 102. exp *Social Behavior/ |
| 103. ((behavio?ral adj (conduct or disorder*)) or conduct disorder*).tw,kf. |
| 104. exp Aggression/ |
| 105. Interpersonal Relations/ |
| 106. Attention/ |
| 107. Attention Deficit Disorder with Hyperactivity/ |
| 108. concentrat*.tw,kf. |
| 109. Cognitive develop*.tw,kf. |
| 110. Language Development Disorders/ |
| 111. Language Development/ |
| 112. Communication/ |
| 113. Speech Perception/ |
| 114. Verbal Behavior/ |
| 115. Vocabulary/ |
| 116. exp Self Concept/ |
| 117. (self-esteem or self esteem).tw,kf. |
| 118. Self Efficacy/ |
| 119. Self-Control/ |
| 120. (self regulation or self control).tw,kf. |
| 121. Executive function/ |
| 122. exp Memory/ |
| 123. Depression/ |
| 124. exp Mood Disorders/ |
| 125. exp Anxiety Disorders/ |
| 126. Affective Symptoms/ |
| 127. Stress, Psychological/ |
| 128. "Quality of Life"/ |
| 129. Temperament/ |
| 130. (depression or depressive).tw,kf. |
| 131. "Growth and Development"/ |
| 132. exp Growth/ |
| 133. exp Child Development/ |
| 134. growth.tw,kf. |
| 135. exp Wounds/ and Injuries/ |
| 136. Accidents/ |
| 137. exp Accidental Falls/ |
| 138. Accidents, Home/ |
| 139. exp Drowning/ |
| 140. exp Emotions/ |
| 141. emotional regulation.mp. |
| 142. well-being.mp. |
| 143. or/19-142 |
| 144. 18 and 143 |
| 145. remove duplicates from 144 |

**EMBASE**

| 1. Sleep/ |
| --- |
| 2. Sleep Time/ |
| 3. (sleep adj3 duration).tw. |
| 4. exp Sleep Disordered Breathing/ and (apnea or apnoea).ti. |
| 5. (or/1-3) not 4 |
| 6. Polysomnography/ |
| 7. Accelerometer/ or Accelerometry/ |
| 8. Actigraphy/ |
| 9. (polysomnogr* or actigr* or accelerom*).tw. |
| 10. (Sleep* adj3 (report or questionnaire* or index or eval* or diary or diaries or log* or journal*)).tw. |
| 11. (self report* or proxy report*).tw. |
| 12. ((objectiv* adj3 measur*) or (direct* adj3 measure*)).tw. |
| 13. or/6-12 |
| 14. 5 and 13 |
| 15. limit 14 to (infant or preschool child <1 to 6 years>) |
| 16. 14 and (pre-school* or preschool* or early childhood).tw,kw. |
| 17. 15 or 16 |
| 18. limit 17 to (english or french) |
| 19. limit 18 to embase |
| 20. remove duplicates from 19 |
| 21. sedentary lifestyle/ |
| 22. (sedentary or inactiv* or (lack adj2 activity)).tw,kw. |
| 23. ((low adj3 energy expend*) or physical* inactiv*).tw,kw. |
| 24. ((chair or stroller or car or automobile* or auto or motor vehicle* or bus or indoor* or in-door or computer) adj3 time).tw,kw. |
| 25. sitting.tw,kw. |
| 26. bed rest.mp. |
| 27. television viewing/ or television/ or exp computer/ |
| 28. internet/ |
| 29. Social Media/ or Mobile Application/ or Mobile Phone/ |
| 30. (screen based entertainment or screen time).tw. |
| 31. (texting or text messag* or app or apps or mobile applications).tw. |
| 32. (smartphone* or smart phone* or cell phone* or mobile phone* or small screen*).tw. |
| 33. (iphone* or ipad* or ipod* or tablet* or laptop*).tw. |
| 34. (social media or Facebook or Youtube or Twitter or Snapchat or Instagram or Pinterest or Skype or Vine).tw. |
| 35. exp physical activity/ or exp exercise/ or exp kinesiotherapy/ or physical education/ or exp sport/ |
| 36. (sport* or bicycl* or swim* or walk* or run* or jog*).tw,kw. |
| 37. (aerobic adj2 (train* or active*)).tw,kw. |
| 38. Play/ and (activ* or outdoor*).tw,kw. |
| 39. (((activ* or outdoor*) adj3 play*) or playground*).tw,kw. |
| 40. active.ti. and (space* or behavio?r* or transport* or commut* or neighbo?rhood* or park* or game* or gaming or lifestyle).mp. |
| 41. (active adj3 (space* or behavio?r* or transport* or commut* or neighbo?rhood* or park* or game* or gaming or lifestyle)).tw,kw. |
| 42. (prone position* or floor time).tw,kw. |
| 43. ((abdomen or stomach or tummy or belly) adj2 (time or play)).tw,kw. |
| 44. exp obesity/ or exp adipose tissue/ or body composition/ or body fat/ or body fat distribution/ or skinfold thickness/ or exp body weight/ or body mass/ |
| 45. (obes* or overweight or over-weight or adipos* or fat or waist or skin fold* or skinfold* or body composition* or bmi or body mass index).tw,kw. |
| 46. dual energy x ray absorptiometry/ or photon absorptiometry/ |
| 47. (bio-impediance analysis or bia or absorptiomet* or densitometr* or photodensitometr* or DXA or DEXA).tw,kw. |
| 48. (physical conditioning or fitness).tw,kw. |
| 49. metabolic syndrome X/ or insulin resistance/ or non insulin dependent diabetes mellitus/ or glucose blood level/ or glucose intolerance/ or exp glucose tolerance test/ or hemoglobin A1c/ |
| 50. ((metabolic adj2 syndrome) or syndrome x).tw,kw. and Risk Factor/ |
| 51. HbA1c.tw,kw. |
| 52. exp cardiovascular disease/ or ((cardiovascular or heart or vascular) adj2 risk*).tw,kw. |
| 53. exp hypertension/ or exp blood pressure measurement/ or exp blood pressure/ or hyperlipidemia/ or exp hypertriglyceridemia/ |
| 54. (hypertens* or high blood pressure or cholesterol or hypercholester* or hyperlipid* or dyslipid*).tw,kw. |
| 55. exp psychomotor performance/ |
| 56. exp motor activity/ |
| 57. (motor development or gross motor skill* or fine motor skill* or locomotor control or object control).tw,kw. |
| 58. exp developmental disorder/ or exp child development/ or developmental milestone.tw,kw. |
| 59. exp behavior disorder/ or child behavior/ or adaptive behavior/ or exp aggression/ or exp *social behavior/ or human relation/ |
| 60. (prosocial behav* or social behav* or ((behavio?ral adj (conduct or disorder*)) or conduct disorder*)).tw,kw. |
| 61. attention deficit disorder/ or exp attention/ or cognitive development/ or achievement/ or exp academic achievement/ or educational status/ |
| 62. (concentrat* or academic achievement or educational achievement or grade-point average or grade point average or GPA).tw,kw. |
| 63. exp developmental language disorder/ |
| 64. language development/ |
| 65. exp interpersonal communication/ |
| 66. speech perception/ |
| 67. exp verbal behavior/ |
| 68. exp developmental language disorder/ or language development/ or exp interpersonal communication/ or speech perception/ or exp verbal behavior/ |
| 69. exp self concept/ |
| 70. (self esteem or self esteem or self regulation or self control).tw,kw. |
| 71. executive function/ or exp memory/ or exp mood disorder/ or exp anxiety disorder/ or emotional disorder/ or mental stress/ or exp "quality of life"/ or exp temperament/ |
| 72. (depression or depressive).tw,kw. |
| 73. "growth, development and aging"/ |
| 74. growth/ or body growth/ |
| 75. Child Development/ |
| 76. Growth.tw,kw. |
| 77. exp injury/ |
| 78. "accidents and accident related phenomena"/ or accident/ or accident proneness/ |
| 79. emotionality/ |
| 80. emotional regulation.mp. |
| 81. wellbeing/ |
| 82. or/21-81 |
| 83. 20 and 82 |
| 84. limit 83 to conference abstract |
| 85. 83 not 84 |

**PsycINFO**

| 1. Sleep/ or Sleep Deprivation/ |
| --- |
| 2. (sleep adj3 duration).tw. |
| 3. *Sleep Apnea/ and (apnea or apnoea).ti. |
| 4. (1 or 2) not 3 |
| 5. Polysomnography/ |
| 6. Monitoring/ |
| 7. (activPAL* or ActiGraph* or acceleromet* or heartrate monit* or heart rate monit* or pedomet* or armband* or arm band* or inclinomet*).tw. |
| 8. (activity monitor* or activity tracker* or fitness tracker* or portable monitor* or wearable monitor* or Fitbit* or Vivofit* or Fuelband*).tw. |
| 9. ((objectiv* adj3 measur*) or (direct* adj3 measure*)).tw. |
| 10. Self report/ or Questionnaires/ or Reproducibility of Results/ |
| 11. Self Monitoring/ |
| 12. Journal Writing/ |
| 13. (report* or self-report* or questionnaire* or diary or diaries or scale* or interview* or journal*).tw. |
| 14. or/5-13 |
| 15. 4 and 14 |
| 16. sedentar*.tw,id. |
| 17. ((low adj3 energy expend*) or physical* inactiv*).tw,id. |
| 18. ((chair or stroller or car or automobile* or auto or motor vehicle* or bus or indoor* or in-door or computer) adj3 time).tw,id. |
| 19. sitting.tw,id. |
| 20. ((television adj watch*) or tv watch* or cartoon*).tw,id. |
| 21. television viewing/ |
| 22. (television or screentime or ((screen or computer) adj3 time) or ((watch* or view*) adj2 (dvd* or video*)) or screen media or social media or video gam* or videogam* or computer gam* or electronic gam* or gaming).tw,id. |
| 23. screen based entertainment.tw,id. |
| 24. exp Social Media/ |
| 25. exp Mobile Devices/ |
| 26. (smartphone* or smart phone* or cell phone* or mobile phone* or small screen*).tw,id. |
| 27. (texting or text messag* or app or apps or mobile applications).tw,id. |
| 28. (iphone* or ipad* or ipod* or tablet* or laptop*).tw,id. |
| 29. bed rest.tw,id. |
| 30. physical activity/ or exp exercise/ or activity level/ or movement therapy/ or dance therapy/ or mind body therapy/ or energy expenditure/ or physical education/ or exp sports/ |
| 31. (sport* or bicycl* or swim* or walk* or run* or jog*).tw,id. |
| 32. (physical* adj2 activ*).tw,id. |
| 33. (aerobic adj2 (train* or active*)).tw,id. |
| 34. (childhood play behavior/ or childhood play development/ or games/ or recreation/) and (activ* or outdoor*).tw,id. |
| 35. ((activ* or outdoor*) adj3 play*).tw,id. |
| 36. playgrounds/ or playground*.tw,id. |
| 37. active.ti. and (space* or behavio?r* or transport* or commut* or neighbo?rhood* or park* or game* or gaming or lifestyle).tw,id. |
| 38. (active adj3 (space* or behavio?r* or transport* or commut* or neighbo?rhood* or park* or game* or gaming or lifestyle)).tw,id. |
| 39. (prone position* or floor time).tw,id. |
| 40. ((abdomen or stomach or tummy or belly) adj2 (time or play)).tw,id. |
| 41. exp overweight/ or body weight/ or weight gain/ or weight loss/ or body fat/ or body mass index/ or weight control/ or body size/ |
| 42. (obes* or overweight or over-weight or adipos* or fat or waist or skinfold* or skin fold* or body composition or bmi or body mass index).tw,id. |
| 43. (bio-impedance analysis or BIA or absorptiomet* or densitometr* or photodensitometr* or DXA or DEXA).tw,id. |
| 44. physical fitness/ or physical endurance/ or physical strength/ or "range of motion"/ |
| 45. (physical conditioning or fitness).tw,id. |
| 46. metabolic syndrome/ or insulin/ or blood suger/ or type 2 diabetes/ or glucose metabolism/ or glucose/ |
| 47. ((metabolic adj2 syndrome) or syndrome x).tw,id. and Risk Factors/ |
| 48. (glycosylated hemoglobin A or HbA1c).tw,id. |
| 49. exp Cardiovascular Disorders/ or exp hypertension/ or cholesterol/ or lipids/ |
| 50. ((cardiovascular or heart or vascular) adj2 risk*).tw,id. |
| 51. (hypertens* or high blood pressure or cholesterol or hypercholester* or hyperlipid* or dyslipid*).tw,id. |
| 52. exp motor development/ or exp motor processes/ |
| 53. (motor development or motor skill* or locomotor control or object control).tw,id. |
| 54. injuries/ or exp head injuries/ or exp spinal cord injuries/ or exp wounds/ |
| 55. developmental disabilities/ or exp delayed development/ or exp intellectual development disorder/ |
| 56. developmental milestone*.tw,id. |
| 57. behavior disorders/ or exp aggressive behavior/ or exp antisocial behavior/ or exp behavior problems/ |
| 58. exp social behavior/ |
| 59. interpersonal relationships/ or exp interpersonal interaction/ |
| 60. ((behavio?ral adj (conduct or disorder*)) or conduct disorder* or prosocial behav* or social behav*).tw,id. |
| 61. exp attention/ or exp attention deficit disorder/ or concentration/ or distraction/ or concentrat*.tw,id. |
| 62. exp cognitive development/ |
| 63. exp academic achievement/ or academic achievement prediction/ or academic aptitude/ or academic failure/ or educational attainment level/ or achievement/ or student admission criteria/ or educational measurement/ |
| 64. (cognitive develop* or academic achievement or educational achievement or grade-point average or grade point average or GPA).tw,id. |
| 65. exp Language Development/ or exp Language Disorders/ or exp communication/ or speech perception/ or vocabulary/ |
| 66. self-perception/ or exp self-concept/ or self-efficacy/ or self-esteem/ or self-control/ or anger control/ or exp emotional control/ or emotional regulation/ or exp impulse control disorders/ or self-regulation/ |
| 67. (self-esteem or self esteem or self regulation or self control).tw,id. |
| 68. exp executive function/ or exp cognitive ability/ or exp memory/ or exp affective disorders/ or exp anxiety disorders/ or psychological stress/ or "quality of life"/ or exp Life Satisfaction/ or exp Well Being/ or personality/ |
| 69. (depression or depressive).tw,id. |
| 70. Development/ |
| 71. exp Childhood Development/ |
| 72. Growth.tw,id. |
| 73. exp Accidents/ or exp Accident Proneness/ or exp Injuries/ |
| 74. Emotional Regulation/ |
| 75. exp Emotional Control/ |
| 76. Well Being/ |
| 77. or/16-76 |
| 78. 15 and 77 |
| 79. limit 78 to (140 infancy <2 to 23 mo> or 160 preschool age ) |
| 80. 78 and (pre-school* or preschool* or early childhood).mp. |
| 81. 79 or 80 |
| 82. limit 81 to (english or french) |
| 83. limit 82 to ("erratum/correction" or journal article) |
| 84. 83 or (82 and retraction.ti.) |
| 85. remove duplicates from 84 |

**CENTRAL**

| 1. sleep.tw,kw. |
| --- |
| 2. 1 and (infant* or child* or preschool* or pre-school*).tw,kw. |
| 3. limit 2 to medline records |
| 4. limit 2 to embase records |
| 5. 2 not (3 or 4) |
| 6. remove duplicates from 5 |
| 7. 6 not (apnea or apnoea).ti. |
