# Supplementary material for: Systematic review of the relationships between sleep duration and health indicators in the early years (0–4 years)
Source: BMC Public Health. 2017 Nov 20;17(Suppl 5):855. doi: 10.1186/s12889-017-4850-2 (PMC5773910; doi:10.1186/s12889-017-4850-2)
Supplement: Supplementary file 2 — Summary of studies included in the systematic review sorted by (whenever possible) outcome indicator, study design, age group, and sleep assessment (objective, then subjective). (DOC 254 kb) [file 12889_2017_4850_MOESM2_ESM.doc]

**Additional File 2: Table S2.**

Summary of studies included in the systematic review sorted by outcome indicator, study design, age group and sleep assessment (objective then subjective) whenever possible.

| ***Outcome: Adiposity*** | | | | | | |
| --- | --- | --- | --- | --- | --- | --- |
| Reference; Country; Large Study/Cohort Name | Study Design | Sample | Exposure | Outcome | Main Findings | Covariates |
| Agras et al. [17]  USA | Longitudinal | N=150  Age: From birth to 9.5 years of age | **Sleep duration** was assessed annually by parent reports at ages 2 through 5 years. This measure was stable from 3 to 5 years, which data were averaged. The 2-year data were not stable and were entered separately. | BMI (from measured height and weight) was used to define **overweight/obesity** (CDC criteria) at 9.5 years of age. | Hours of sleep reported annually from 3 through 5 years were negatively related with overweight/obesity; children who were to become overweight/obese slept about 30 min less on average than those who would remain normal weight. This difference was almost entirely due to shorter daytime sleep, with only 5 minutes’ difference in nighttime sleep. | None |
| Reilly et al. [18]  UK  Avon Longitudinal Study of Parents and Children | Longitudinal | N=5,493  Age: Birth cohort followed until 7 years of age | **Duration of nighttime sleep** at 38 months was parent-reported in a questionnaire. Quartiles of sleep duration were created: <10.5 h, 10.5-11.4 h, 11.5-11.9 h, and ≥12 h per night. | BMI (from measured height and weight) was used to define **obesity** (BMI ≥95^th^ centile relative to reference data for the UK population in 1990) at 7 years of age. | Sleep duration in children aged 3 years was independently associated with prevalence of obesity at age 7. Children in the lowest two quarters of sleep duration (<10.5 h/night; OR=1.45, 95% CI 1.10-1.89, and 10.5-10.9 h/night; OR=1.35, 95% CI 1.02-1.79) were more likely to be obese at age 7 than children in the highest quarter (>12 h/night). | Maternal education, energy intake at age 3 years (food groups) and sex |
| Lampl et al. [29]  USA | Longitudinal | N=23  Age: 4-17 months | Daily parental diaries continuously recorded **sleep duration** over 4 to 17 months. | **Weight**, **weight-for-length ratio**, and **3 skinfold variables** (upper arm, thigh, and calf), trunk (subscapular and abdominal), and abdominal (abdominal and suprailiac) subcutaneous adiposity. | Increased sleep bout duration predicted weight (p<0.001) and abdominal skinfold accrual (p=0.05) contingent on length growth, and truncal adiposity independent of growth (p<0.001). | Age (for weight gain and abdominal skinfolds) and age, sex, weight and concomitant length growth (for trunk skinfolds) |
| Bonuck et al. [19]  England  Avon Longitudinal Study of Parents and Children | Longitudinal | N=1,899  Age: Sleep was determined at ages 18 months, 2.5 years, 4.75 years, 5.75 years, and 6.75 years | **Sleep duration** was calculated from maternal report of typical weekday bed- and wake-times. | **Obesity** using the IOTF definition and from measured height and weight. | Children with short sleep duration at 4.75 years (≤10.5 h) were more likely to be obese at 15 years compared to those sleeping >10.5 h and <12.1 h (OR=2.04, 95% CI 1.36-3.04). Longitudinal associations with the other time points of sleep duration were not significant. | Sex, age, child’s estimated weight and height at 6 months, maternal education, parity and prepregnancy BMI, tonsillectomy and adenoidectomy, and sleep-disordered breathing cluster |
| Touchette et al. [20]  Canada  Quebec Longitudinal Study of Child Development | Longitudinal | N=1,138  Age: 2.5 to 6 years old | **Sleep duration** was reported at 2.5, 3.5, 4, 5, and 6 years based on the last month by an open question from the Self-Administered Questionnaire for Mother: “Indicate how long in total your child sleeps during the night (on average). Do not count the hours that your child is awake.” Four sleep duration patterns were identified: short persistent, short increasing, 10-h persistent, and 11-h persistent. | BMI (from measured height and weight) was used to define **overweight/obesity** (IOTF definition) at 2.5 and 6 years old. | The risk of being overweight or obese was 4.2 times higher for short (<10 h) persistent sleepers (OR=4.2, 95% CI 1.6-11.1) and 1.8 times higher for 10-h persistent sleepers (OR=1.8, 95% CI 1.1-2.9) than for 11-h persistent sleepers. | Birth weight, prematurity, low birth weight, sex of the child, maternal smoking during pregnancy, weight at 5 months, low parental education, modified family structure, late cereal introduction, not breast-fed, immigrant mother, naptime at 2.5 years, watching TV at 6 years, playing video games at 6 years, doing physical activities, overeating at 6 years, snacking at 6 years, eating sweets at 6 years, snoring at 6 years, and low income status at 6 years |
| Speirs et al. [21]  USA  STRONG Kids study | Longitudinal | N=247  Age: 38.0 months old at T1 (1-y follow-up) | Mothers responded to the following question about children’s **sleep duration**: “During the past week, how many hours of sleep did your child get each night (on average)?” | Child **BMI percentile** was obtained from measured height and weight and using the CDC criteria. | Shorter child nighttime sleep was associated with higher BMI percentile at T2 (β = -7.31, p<0.001). | Child’s sex and age, and mother’s sex, age, education, marital status, race/ethnicity, annual household income, and maternal BMI |
| Magee et al. [22]    Australia  Longitudinal Study of Australian Children | Longitudinal | N=2,984  Age: Children were followed from 4 to 5 years of age until 8 to 9 years of age | Time-use diaries were used to assess **sleep duration**. At each of 3 waves, the parent completed two diaries; one encompassed a 24-h period on a weekday, the other a 24-h period on a weekend day. | **BMI** (kg/m^2^) was calculated from measured height and weight at each wave. | Short sleep duration at 4 to 5 years of age was significantly associated with higher BMI at 8 to 9 years of age (β=-0.07, p=0.04). | Sex, sleep problems, household income, maternal education, maternal weight status |
| Hiscock et al. [27]  Australia  Longitudinal Study of Australian Children | Cross-Sectional and Longitudinal | N=7,701  Mean age: Infant cohort: 8.7 months; Preschool cohort: 56.8 months. Infants aged 0-1 years (T1) were followed at age 2-3 years (T2) while children aged 4-5 years (T1) were followed at age 6-7 years (T2). | **Sleep duration** was measured using the Children’s Light Time-Use Diary (two 24-h diaries). | **BMI z-scores** (from measured height and weight and the CDC definition) and **BMI status** (underweight, normal weight, overweight, obese; using the IOTF definition) were used in this study. | Cross-sectionally: Obesity was not associated with sleep duration at 0-1, 2-3 or 4-5 years.  Longitudinally: Sleep duration at 0-1 year did not predict BMI z-score at 2-3 years. Sleep duration at age 4-5 years did not predict BMI z-score at age 6-7 years. | None |
| Klingenberg et al. [28]  Denmark  SKOT cohort | Cross-Sectional and Longitudinal | N=311  Age: 9 months, 18 months, and 3 years of age | Parent-reported **sleep duration** was obtained at 9 months, 18 months and 3 years of age. Additionally, accelerometer- estimated **nocturnal sleep duration** was obtained at 3 years of age (Actigraph GT3X worn over a 7-day period). | **BMI z-scores** (from measured height and weight and the WHO definition), sum of **skin-fold thicknesses** (mm), and **fat mass** (kg and %) from DEXA. | There was no association between sleep duration and adiposity indicators both cross-sectionally and longitudinally. | Birth weight, gestational age, duration of breastfeeding, maternal smoking during pregnancy, maternal BMI at 9 months of investigation, household income, highest education level, and physical activity level (cross-sectional model) |
| Diethelm et al. [23]  Germany  DONALD Study | Cross-Sectional and Longitudinal | N=481  Age: Age 2 until age 7 | At each follow-up visit, the study pediatrician asked the parents: “How many hours does your child usually sleep per day?” **Average daily sleep duration** (h) encompasses day and nighttime sleep. Using median splits of sleeping time at 1.5 and 2 years they defined sleep duration categories as follows: consistently short (CS, n=122), inconsistent (I, n=143), and consistently long (CL, n=216). The 50^th^ percentile of sleep duration was 13 hours at both assessments. | From the age of 2 years onwards they assessed **BMI** (from measured height and weight), **skinfold thicknesses** (biceps, triceps, subscapular, and suprailiac sites), **% overweight/obese** (IOTF definition), and **fat mass index** (fat mass/m^2^; Deurenberg equations). | Cross-sectionally: Excess body fat (%) and fat mass index (kg/m^2^) at age 7 were significantly higher in the CS-group.  Longitudinally: BMI trajectories of CS- or I-sleepers did not differ significantly in the linear, quadratic or cubic trend when compared to CL-sleepers. However, compared to CL-children, CS-sleepers differed in their fat mass index development with respect to linear, quadratic and cubic trend (all p<0.04), resulting in progressively higher fat mass index levels until age 7 independently of early life of socioeconomic factors. Additionally, multivariate adjusted OR for the risk of having excess body fat at age 7 years was significantly higher (p=0.03) for the CS-sleepers (OR=2.4, 95% CI 1.3-4.6) compared to the CL-sleepers. | Sex, birth year, birth weight, rapid weight gain |
| Carter et al. [24]  New Zealand  FLAME study | Cross-Sectional and Longitudinal | N=244  Age: 5 time points: 3, 4, 5, 6, and 7 years old. Children were followed from age 3 to 7 in the longitudinal sample. | **Sleep duration** was measured using Actical accelerometers (Mini-Mitter) for 5 consecutive days, including two weekend days. | **BMI** (from measured height and weight), **fat mass index** (from bioelectrical impedance and DEXA) and **%OW/OB** (CDC definition) were used in this study. | Cross-sectionally: Sleep duration was not significantly associated with adiposity indicators after adjustment for covariates.  Longitudinally: After adjustment for multiple confounders, each additional hour of sleep at ages 3-5 was associated with a reduction in BMI of 0.48 (95% CI 0.01 to 0.96), a reduction of FMI of 0.48 (0.10 to 0.86) and a reduced risk of being overweight/obese of 0.39 (0.24 to 0.63) at age 7. | Age, sex, maternal education, maternal BMI, income, ethnicity, birth weight, smoking during pregnancy, physical activity, TV viewing, fruit-vegetable intake and non-core foods intake |
| Butte et al. [25]  USA | Cross-Sectional and Longitudinal | N=119 (cross-sectional) and N=111 (longitudinal)  Mean age: 4.6 y | **Sleep duration** was measured using an ActiGraph GT3X+ for at least 4 valid days (measurements at baseline and at 1-year follow-up). | Adiposity indicators (**weight, BMI, FFM, FM** and **%FM** were measured by DEXA). | Sleep duration was not associated with adiposity indicators in the cross-sectional sample (p>0.46). However, sleep duration inversely predicted changes in FM (p=0.005) and %FM (p=0.006) in the longitudinal sample. | Cross-sectional sample: age, sex, race/ethnicity, daycare hours, household size, household income, mother’s age, BMI and education. Longitudinal sample: age, sex, race/ethnicity, delta height, daycare hours, household size, household income, mother’s age, BMI and education. |
| Scharf and DeBoer [26]    USA  Early Childhood Longitudinal Study - Birth Cohort | Cross-Sectional and Longitudinal | N=10,700  Age: Children were tested at 4 and 5 years | The primary caregiver (most often the mother) completed a computer-assisted interview at home by trained assessors. **Short sleep duration** was defined as greater than one standard deviation from the mean for the group. | **BMI z-scores** were calculated from measured height and weight. The CDC cut-points were used to define obesity. | Cross-sectionally: Sleep duration at 4 and 5 years was inversely associated with BMI z-scores (p<0.001). Odds of obesity were higher at 4 years for children sleeping <9.44 h/night compared to those sleeping ≥9.44 h/night (adjusted OR 1.36, 95% CI 1.03-1.80). Longitudinally: short sleep duration at 4 years was associated with increases in BMI z-scores between 4 and 5 years (p<0.05). | Sex, race/ethnicity, SES, TV viewing |
| Gibson et al. [38]  New Zealand | Cross-Sectional | N=52  Age: 11-13 months | Infant **sleep duration** was assessed by one week’s actigraphy worn on the calf (AW64 actiwatch, Respironics and Mini Mitter Inc), by sleep diaries and by the Brief Infant Sleep Questionnaire. | Infant’s weight and length were measured by the researcher at study onset and **BMI** (kg/m^2^) was calculated. | Sleep duration was not associated with BMI (non-significant results were not reported). | None |
| Fisher et al. [39]  UK  Gemini Study | Cross-Sectional | N=1,303  Mean age: 15.7 months | **Sleep duration** was assessed using modified items from the Brief Infant Sleep Questionnaire for Children. Participants were then classified into 5 groups: <10 h/night, 10 to <11 h/night, 11 to <12 h/night, 12 to <13 h/night, and ≥13 h/night. | **Weight** was objectively measured and weight standard deviation scores were obtained using UK reference data. | The association between sleep duration and weight was not significant at this age (p=0.13). | None |
| Hager et al. [30]  USA | Cross-Sectional | N=240  Mean age: 20.2 months | Mothers completed the Brief Infant Sleep Questionnaire to assess **nighttime sleep duration.** | Weight-for-length z-scores were obtained (CDC criteria) and **weight status** was reported as a dichotomous variable (obese, ≥95^th^ percentile, and healthy weight, <95^th^ percentile). | Healthy weight toddlers slept 0.7 hours longer/night compared with obese toddlers (p=0.006). In multivariable models, obese toddlers had a significantly shorter nighttime sleep duration compared with healthy weight toddlers (OR=0.69, p=0.01). | Age, sex, maternal marital status, and recruitment location |
| Kuzik and Carson [41]  Canada | Cross-Sectional | N=100  Mean age: 38.5 months | **Total nighttime sleep** was assessed by asking parents: “How long does your child usually sleep per night at the moment?” Responses for hours and minutes were used to calculate an hours/night variable. | **BMI z-scores** (from measured height and weight) were used as an indicator of adiposity (WHO criteria). | Nighttime sleep duration was significantly associated with BMI z-scores (β=0.22, 95% CI 0.05-0.39, p<0.05). | Age, sex, parental education |
| Plancoulaine et al. [31]  France  EDEN Study | Cross-Sectional | N=1,028  Age: 3 years of age | A parental self-administered questionnaire was used to assess **sleep duration**. Short sleepers were defined as children with total sleep duration of less than 12 h per day. | **BMI z-scores** (from measured height and weight) were used as an indicator of adiposity (WHO criteria). | Short sleep duration was associated with higher BMI z-scores (OR: 1.31, 95% CI 1.01-1.69 per additional standard deviation) in boys only. | Centre, familial income, educational level, child-care arrangement, isolated mother, maternal BMI, maternal depression, night awakenings, parental presence when falling asleep, physical activity, TV watching, food patterns |
| Dev et al. [32]  USA  STRONG Kids program | Cross-Sectional | N=407  Age range of 2-5 years | **Sleep duration** was collected from a self-administered questionnaire completed by the child’s parent/primary caregiver. | **BMI** (kg/m^2^) calculated from measured height and weight. **Overweight** and **obesity** were defined using the CDC criteria. | Nighttime sleep duration was negatively associated with child overweight/obesity. Children who slept for 8 hours or less were 2.2 times more likely to be overweight/obese (95% CI 1.3-3.7) compared to those sleeping 9 and more hours. | None |
| Jones et al. [33]  USA  STRONG Kids study | Cross-Sectional | N=337  Mean age: 3.22 y | Parents responded to the following question about children’s sleep: “During the past week, how many hours of sleep did your child get each night (on average)?” Child **sleep hours** were then broken into a dichotomous variable representing children who obtained 10 or more hours of sleep vs. those that obtained less than 10 h of sleep on an average night. | Child **overweight/obesity** status was obtained using the CDC definition from measured height and weight. | Odds of overweight/obesity were 2.87 times higher in children sleeping <10 h/day compared to those sleeping ≥10 h/day (OR=2.87, 95% CI 1.50-5.49). | Parent gender, parent age, racial/ethnic group, family income, parent education, single-parent household, parent BMI |
| Jiang et al. [34]  China  Shanghai Preschool Children Sleep Study | Cross-Sectional | N=1,311  Age: 3.77 y for boys and 3.75 y for girls | Parents filled out a questionnaire about their children’s sleep duration. Parents were asked about their children’s sleep during a typical recent week. Information collected included children’s bedtime and nighttime sleep end time. **Nighttime sleep duration** (grouped as <9 h, 9.0 to 9.4 h, 9.5 to 9.9 h, 10 to 10.4 h, 10.5 to 10.9 h, and ≥11 h) was calculated as nighttime sleep end time minus bedtime. | BMI (from measured height and weight) was used to define **obesity** (BMI ≥95^th^ percentile for the children). | Compared with children reporting ≥11 h of sleep per night, the odds ratio for childhood obesity was 4.76 (95% CI 1.28-17.69) for children with <9 h of sleep, and 3.42 (95% CI 1.12-10.46) for children with 9.0 to 9.4 h of sleep. | Age, sex, appetite, birth weight, mother’s age at delivery, parental and maternal education, geographical area, and household income |
| Sijtsma et al. [35]  Netherlands  GECKO Drenthe Cohort | Cross-Sectional | N=759  Mean age: 3.9 y | Bedtimes and wake times were reported by parents and mean **sleep duration** per night was calculated. | **BMI** (kg/m^2^) was calculated from measured height and weight. | Sleep duration was negatively associated with BMI (r=-0.11, p<0.01). | None |
| Watanabe et al. [36]  Japan | Cross-Sectional | N=1,765  Mean age: 4.2 y | A self-administered questionnaire was filled out by the principal caregiver to assess sleeping habits. Sleeping habits included wake time and bedtime on weekdays and weekends. **Nighttime sleep duration** per day was calculated as wake time minus bedtime, summing the total hours for the week, and dividing by 7 days. Sleep duration was then dichotomized at <10 h per day or ≥10 h per day. | BMI (from measured height and weight) was calculated and children were categorized as **overweight/obese** based on the IOTF criteria. | Children sleeping <10 h/day had higher odds of being overweight/obese compared to those sleeping ≥10 h/day (OR=1.96, 95% CI 1.28-3.01). | Age, sex, birth weight, number of siblings, parental overweight/obesity, maternal employment status and type of family household |
| Dieu et al. [37]  Vietnam | Cross-Sectional | N=670  Mean age: 56.2 months | **Sleep duration** information was collected using an interview-administered, pre-coded questionnaire. The average amount of time spent sleeping at night was analyzed as a continuous variable. | BMI (from measured height and weight) was used to define **overweight/obesity** (IOTF definition). | Children who had longer duration of sleep had a significant decrease by 13% (PR=0.87, 95% CI 0.78-0.98) in the odds of being overweight/obese for each additional hour of sleep. This association was even stronger for obesity alone as the outcome measure (PR=0.75, 95% CI 0.60-0.94). | Gender, father’s education level, parental BMI status, household wealth index, birth weight, and duration of breastfeeding |
| Cardon et al. [40]  International - Belgium, Bulgaria, Germany, Greece, Poland, Spain  ToyBox-study | Cross-Sectional | N=3,301  Mean age: 4.7 y | The Primary Caregivers’ Questionnaire was used to assess **sleep duration** on weekdays and weekends. The cut-off value of 11 hours/night was used to dichotomize sleep duration. | BMI (from measured height and weight) and **overweight/obesity** (IOTF definition) were used as outcome measures. | Sleep duration was not significantly associated with adiposity in this cohort. Boys and sleep duration on weekdays (h/night): 9.98 (1.22) in normal-weight boys vs. 9.87 (1.21) in overweight/obese boys, NS. With BMI: Beta = 0.023 (95% CI -0.047; 1.030; p=0.53). OR for <11h/night vs. ≥11 h/night for OW/OB: 0.79 (95% CI 0.37-1.67, p=0.53). Boys and sleep duration on weekends (h/night): 10.38 (1.31) in normal-weight boys vs. 10.19 (1.20) in overweight/obese boys, NS. With BMI: Beta = -0.057 (95% CI -0.130; 0.006, p=0.10). OR for <11h/night vs. ≥11 h/night for OW/OB: 1.62 (95% CI 0.92-2.85, p=0.09). Girls and sleep duration on weekdays (h/night): 10.05 (1.16) in normal-weight girls vs. 9.72 (1.25) in overweight/obese girls, NS. With BMI: Beta = -0.015 (95% CI -0.106; 0.067; p=0.70). OR for <11 h/night vs. ≥11 h/night for OW/OB: 0.93 (95% CI 0.43-2.01, p=0.85). Girls and sleep duration on weekends (h/night): 10.52 (1.24) in normal-weight girls vs. 10.24 (1.30) in overweight/obese boys, NS. With BMI: Beta = -0.043 (95% CI -1.121; 0.035, p=0.24). OR for <11 h/night vs. ≥11 h/night for OW/OB: 1.64 (95% CI 0.98-2.75, p=0.06). | Age, educational level of the mother, and country |
| ***Outcome: Emotional Regulation*** | | | | | | |
| Miller et al. [42]  USA | Cross-Over Trial | N=12  Mean age: 33.9 months | Acute sleep restriction (nap deprivation) protocol. Children slept on a strict schedule (**sleep duration** verified with wrist actigraphy and sleep diaries) for 5 days before each of two afternoon assessments following a nap and a no-nap condition. On average, children lost about 90 min of sleep on the no-nap vs. the nap day. | **Self-regulation strategies** in a challenge context were assessed in a nap vs. no-nap intervention. Children were videotaped while attempting an unsolvable puzzle, and 10 mutually exclusive self-regulation strategies were later coded. | Nap deprivation (therefore reduced total sleep duration) resulted in moderate-to-large effects on self-regulation strategies, with decreases in skepticism (d=0.77; 7% change), negative self-appraisal (d=0.92; 5% change) and increases in physical self-soothing (d=0.68; 10% change), focus on the puzzle piece that would not fit (perseveration; d=0.50; 9% change) and insistence on completing the unsolvable puzzle (d=0.91; 10% change). After losing daytime sleep, toddlers were less able to engage effectively in a difficult task and reverted to less mature self-regulation strategies than when they were well rested. | None |
| Berger et al. [43]  USA | Cross-Over Trial | N=10  Mean age: 34 months | Acute sleep restriction (nap deprivation) protocol. Children followed a strict sleep schedule (≥12.5 h time in bed per 24-h) for 5 days, before each of two randomly assigned afternoon emotion assessments following Nap and No-Nap conditions (resulting in an 11-day protocol). Actigraphy (model AW64) was used to measure **sleep duration** in this study. Parents also completed a sleep diary on each study day. | **Emotion responses** were assessed using an emotion-elicitation and a challenge protocol. Children viewed emotion-eliciting pictures (five positive, three neutral, three negative) and completed puzzles (one solvable, one unsolvable). Children’s faces were video-recorded, and emotion displays were coded. | When sleep restricted, children displayed less confusion in response to neutral pictures, more negativity to neutral and negative pictures, and less positivity to positive pictures. Sleep restriction also resulted in a 34% reduction in positive emotion responses (solvable puzzle), as well as a 31% increase in negative emotion responses and a 39% decrease in confused responses (unsolvable puzzle). | None |
| Gribbin et al. [44]  USA | Non-Randomized Intervention | N=7  Age: 2- to 4-year-old children | During a 25-day in-home protocol, researchers collected four salivary cortisol samples (0, 15, 30, 45 min post-wake) following five polysomnographic sleep recordings on non-consecutive days after 4 h (morning nap), 7 h (afternoon nap), 10 h (evening nap), 13 h (baseline night), and 16 h (sleep restriction night) of wakefulness. Polysomnography was performed with a portable Vitaport 3:16 channel EEG recorder (Temec Instruments) according to standard criteria to **assess sleep duration**. Actigraphy (actigraph model AW2, Philips Respironics) worn on the wrist was also used to verify sleep schedule compliance. Finally, parents completed a 26-item daily sleep diary. | **Cortisol awakening response** (CAR) was determined from saliva samples. Saliva cortisol was collected at 4 points after five PSG-recorded sleep episodes (morning nap, afternoon nap, evening nap, baseline night, sleep restriction night): 0 (wake time), 15, 30, and 45 minutes after wake time. One sampling day was carried out per sleep condition. Thus, a total of 20 salivary cortisol samples were collected from each child during the 25-day protocol. | The CAR was robust after nighttime sleep, diminished after sleep restriction, and smaller but distinct after morning and afternoon (not evening) naps. Cortisol remained elevated 45 min after morning and afternoon naps. | None |
| Jansen et al. [45]  Netherlands  Generation R Study | Longitudinal | N=4,782  Age: 2 months, 24 months and 36 months | At 2 months and 24 months, a parental questionnaire was used to assess **sleep duration**. Average sleep duration of children per 24 hours was based on the number of sleeping hours during both night and day and quartiles were used for analysis. | The outcome measure was the **Anxious/Depressed syndrome scale** from the Child Behavior Checklist for toddlers when the children were 18 months and 3 years old. The sum score of this scale was dichotomized for analysis (20% highest scores defined as having anxiety or depressive symptoms). | At 2 months of age, sleep duration was not associated with later anxiety or depressive symptoms. At 24 months, however, a relative short sleep duration (lowest quartile; <12.5 h/day) was a risk factor for anxiety or depressive symptoms at the age of 3 years (OR=1.32, 95% CI 1.07-1.62). | Child age, ethnicity, gender, maternal age, maternal education level, civil status, maternal psychopathological symptoms, and Anxious/Depressed syndrome scale at 18 months |
| Saenz et al. [47]  USA | Longitudinal | N = 47  Age: 3- to 4-month-old infants at T1 and 18- to 24-month-old toddlers at T2 | **Sleep duration** was objectively measured using an Actiwatch monitor for 5 days. | **Social-emotional problems** were assessed by the parents using the Brief Infant-Toddler Social and Emotional Assessment. | Sleep duration was not associated with social-emotional problems in boys (all p>0.05). In girls, the only significant association was between sleep duration and autism spectrum behaviors (β=-0.48, p<0.05). | Sleep efficiency |
| Bouvette-Turcot et al. [46]  Canada  MAVAN Study | Longitudinal | N = 209  Age: 6, 12, 18, 24 and 36 months | Questions on **sleep duration** were adapted from the Self-Administered Questionnaire for the Mother. | **Negative emotionality/behavioral dysregulation** was assessed using a composite score derived from the Early Childhood Behavior Questionnaire (ECBQ). | Simple slope results revealed significant average sleep duration effect on child negative emotionality/behavioral dysregulation in children who carry either 1 or 2 copies of the S allele (β=-0.55, p<0.001). | Gender, SES, and maternal depression level |
| Kobayashi et al. [48]  Japan | Longitudinal | N=41,890  Age: 2 y and 8 y | Parents filled out a questionnaire asking questions about morning waking times and bedtimes of their children**. Sleep duration** was calculated as time from going to bed (bedtime) to waking up at the age of 2. When either wake-up time or bedtime was irregular, sleep duration was defined as irregular. | The presence or absence of three attention problems (interrupting people, inability for the child to wait his/her turn during play, and failure to pay attention to the surrounding area when crossing the street) and four aggressiveness problems (lying, destroying toys and/or books, hurting other people, and causing disturbances in public) were assessed at 8 years of age. An outcome of **attention problems** was defined as the existence of all three attention problems, and an outcome of **aggressiveness** was defined as the existence of all four delinquent/aggressive behaviors at age 8 years of age. | An irregular sleep duration was positively correlated with the outcome of aggressive behaviors (OR=1.91, 95% CI 1.23-2.98). However, the associations between sleep duration and attention problems or problems of delinquent/aggressive behaviors were not significant. | Gender, educational attainment of the parents, and the primary caregiver of the child |
| Shinohara and Kodama [49]  Japan | Cross-Sectional and Longitudinal | N=31  Age: 4-6 weeks, 8-10 weeks, and 14-16 weeks  Follow-up: At 4- to 6-week intervals | **Sleep duration** was assessed using an actigraph attached to the left ankle of infants for 3 successive days. | The mothers recorded the duration of crying/fussy behavior of infants in a timetable. The mean value during the 3-day period was defined as the 24-h **crying/fussy behavior duration** in the infant at each measurement point. | Sleep duration was not associated with 24-h crying/fussy behavior either cross-sectionally (4-6 weeks: r=-0.08, p=0.67; 8-10 weeks: r=-0.01, p=0.96; 14-16 weeks: r=0.03, p=0.88) or longitudinally (r=-0.02, p=0.89). | None |
| Kaley et al. [50]  England | Cross-Sectional | N=74  Age: 4-9 weeks | Data on **infant sleep duration** were collected by parental diaries (mothers) for a minimum of 3 days over the period of a week. | The Early Infancy Temperament Questionnaire was used and the following 9 sub-scale scores were reported: **activity, rhythmicity, approach, adaptability, intensity, mood, persistence, distractibility,** and **threshold.** | Total sleep duration was significantly associated with better “approach” (r=-0.38, p=0.0001), “intensity” (r=-0.30, p=0.008), and “distractibility” (r=-0.23, p=0.038). The associations between total sleep duration and the other sub-scales were not significant. Note: A negative association denotes a favorable association. | None |
| Spruyt et al. [63]  Australia | Cross-Sectional | N=20  Age: 3 months, 6 months, 11 months and 12 months | **Sleep duration** was assessed using both actigraphy worn on the calf (Actiwatch AW64, Mini Mitter Company Inc) and parental sleep diaries over 3 consecutive days. | The Early Infant Temperament Questionnaire (at 3 months) and the Revised Infant Temperament Questionnaire (at 6 and 11 months) were used to assess the following 9 sub-scale scores: **activity, rhythmicity, approach, adaptability, intensity, mood, persistence, distractibility,** and **threshold.** The Bayley Scales of Infant Development II was also used at 12 months to assess **mental,** motor and **behavioral development**. | The reported 24-h sleep duration was correlated with higher approachability at 3 months (r=-0.62, p=0.01), higher rhythmicity at 6 months (r=-0.52, p=0.04) and higher rhythmicity at 11.5 months (r=-0.59, p=0.02). The 24-h sleep duration measured by actigraphy was correlated with higher distractibility at 6 months (r=0.52, p=0.04) and higher persistence at 11.5 months (r=0.61, p=0.01). At 12 months of age diurnal sleep duration, measured by the sleep diary, was negatively correlated with emotional regulation (r=-0.77, p<0.01). In addition, total behavioral scoring decreased when the infant had more diurnal sleep duration, as measured by sleep diary (r=-0.68, p<0.05). | None |
| Mindell and Lee [51]  Brazil | Cross-Sectional | N=1,351  Mean age: 7.4 months | The Brief Infant Sleep Questionnaire was filled out by the mothers and used to assess **sleep duration**. | Mother were asked 3 questions about their perception of their child’s **mood** at bedtime, in the morning, and throughout the day on a 5-point scale. Also, the Ages and Stages Questionnaire was filled out by the mothers to assess **personal social skills**. | Shorter nighttime sleep duration was associated with worse morning mood (r=0.16, p<0.001) and daytime mood (r=0.06, p<0.05) but not with bedtime mood (r=0.04 p>0.05). | None |
| Gibson et al. [59]  New Zealand | Cross-Sectional | N=52  Age: 11-13 months | Infant **sleep duration** was assessed by one week’s actigraphy worn on the calf (AW64 actiwatch, Respironics and Mini Mitter Inc), by sleep diaries and by the Brief Infant Sleep Questionnaire. | The Ages and Stages Questionnaire was completed by parents to assess **personal social skills**. | Sleep duration was not associated with personal social skills (non-significant results were not reported). | Age and gender. |
| Scher et al. [58]  Israel | Cross-Sectional | N=30  Age: 12-month-olds | **Sleep duration** was assessed using an actigraph (Ambulatory Monitoring) attached to the ankle for 2 nights’ recordings. | The Carey Toddler Temperament Questionnaire was completed by the mothers and was used to assess the following categories: **activity, rhythmicity, approach, adaptability, intensity, mood, persistence, distractibility,** and **threshold.** | Sleep duration was not associated with any of the 9 dimensions assessed by the questionnaire (all p>0.05): activity (r=0.29, p>0.10), rhythm (r=0.32, p<0.10), approach (r=0.24, p>0.10), adaptability (r=0.06, p>0.10), intensity (r=0.28, p>0.10), mood (r=0.31, p<0.10), persistent (r=0.27, p>0.10), distractibility (r=-0.07, p>0.10) and threshold (low) (r=-0.10, p>0.10). | None |
| Gibson et al. [59]  New Zealand | Cross-Sectional | N=52  Age: 1-year-old infants | **Sleep duration** of infants was monitored for a week at home using ankle actigraphy (AW64TM actiwatch, Respironics & Mini Mitter Inc) and sleep diaries. | **Mood** was reported by parents using a series of scales included in the diary. These scales provided information on how the infants woke up in the morning; how alert they were; mood in the morning; how active each day had been; how tired they were at bedtime; and the extent of problems putting the infant to sleep in the evening. | Infants rated as being in a good mood in the morning averaged 1.0 h more sleep at night (p<0.05). The other comparisons were not statistically different. | None |
| Scher et al. [52]  Israel | Cross-Sectional | N=31  Mean age: 18.5 months | The Actigraph (Ambulatory monitoring) attached to the child’s leg for two nights was used to assess **sleep duration**. Also, a sleep diary completed by the mother (same two nights) was used to assess sleep duration. | The Carey Toddler Temperament Questionnaire was completed by the mothers to assess the following 9 dimensions: **activity, rhythmicity, approach, adaptability, intensity, mood, persistence, distractibility,** and **threshold.** | Increased rhythmicity was associated with shorter sleep duration (r=0.51, p<0.01). Also, children who were reported to sleep less were perceived by their mothers as less adaptable (r=-0.43, p<0.05) and more distractible (r=-0.37, p<0.05) compared to children who slept longer. | None |
| Hysing et al. [53]  Norway  Akershus Birth Cohort Study | Cross-Sectional | N=2,041  Age: 2 years old | The Brief Infant Sleep Questionnaire was filled out by the mothers and used to assess **sleep duration**. The sleep duration variable was divided into 5 categories: <11 h, 11-12 h, 12-13 h, 13-14 h, ≥14 h. The 13-14 h category was chosen as the reference (largest category). | The Ages and Stages Questionnaire: Social Emotional was filled out by the mothers to assess **social-emotional development**. | Short sleep duration was significantly associated with social-emotional problems in a dose-response manner. For example, sleeping less than 11 h per night was associated with a 5-fold increase in the odds of social-emotional problems compared to sleeping 13-14 h per night (OR=4.98, 95% CI 1.96-12.68). | Maternal age, maternal education, marital status, parity, gestational age, child sex, child birth weight, communication problems, fine motor problems, and gross motor problems |
| Komada et al. [60]  Japan | Cross-Sectional | N=1,746  Age: 2 to 5 years old | Mothers completed a questionnaire on sleep habits of children over a recent typical month and **sleep duration** was determined. **Sleep duration** was divided into short and long groups by the median value (2- to 3-year-old children: 9.5 h; 4- to 5-year-old children: 9.3 h). | Behavioral problems were assessed using the Child Behavior Check List. Behavioral problems included **attention problems, aggressive behavior,** and the **anxious/depressed category**. | The short sleep duration group showed significantly higher aggressive scores than the long sleep duration group among 2- to 3-year-old children. However, the other comparisons were not significantly different. | None |
| Lavigne et al. [54]  USA | Cross-Sectional | N=510  Age: 2 to 5 years | During the interview, the mothers were asked to report the usual time at which their child went to sleep and awoke each day. **Amount of night sleep** was calculated from estimates of usual time to sleep and time to wake up. | Outcome variables included the three-dimensional characteristics of psychopathology (**Child Behavior Checklist Total, Internalizing,** and **Externalizing Problems scores**) and the presence vs. absence of a **DSM-III-R diagnosis** as assigned by psychologists. | The relationship between less sleep at night and the presence of a DSM-III-R psychiatric diagnosis was significant (OR=1.23, p=0.03). Less night sleep (p<0.0001) and less sleep in a 24-h period (p<0.004) were associated with increased total behavior problems on the Child Behavior Checklist; less night sleep (p<0.0002) and less 24-h sleep (p<0.004) were also associated with more externalizing problems on that measure. | None |
| Molfese et al. [61]  USA | Cross-Sectional | N=64  Mean age: 30.14 months | **Sleep duration** was assessed using Actigraphy for at least 4 nights (MicroMini Motion Logger) in addition to a parent-reported sleep diary. | Parents completed the Children’s Behavior Questionnaire - Short Form to assess the following four dimensions: **fear, soothability, approach,** and **activity level.** | Using the sleep diary, total sleep duration was not associated with the dimensions examined. Using actigraphy, total sleep duration was associated with soothability (r=0.28, p<0.05) but not with the 3 other dimensions. | None |
| Keefe-Cooperman and Brady-Amoon [55]  USA | Cross-Sectional | N=874  Mean age: 40.0 months | Parents or caregivers were asked to indicate their child’s **total hours of nighttime sleep** as part of a structured interview protocol. | The Behavior Assessment System for Children, Second Edition (BASC-2) was used to assess behavioral concerns. Three composite scores were used for this study: **internalizing problems, externalizing problems,** and **adaptive skills**. Higher scores on the internalizing and externalizing domains suggest a greater degree of problematic behavior. In contrast, higher scores on the adaptive skills domain suggest higher levels of functioning. | Total nighttime sleep duration was associated with externalizing problems (r=-0.10, p<0.05) and adaptive skills (r=0.09, p<0.05) but not with internalizing problems (r=-0.02, p>0.05). | None |
| Scharf et al. [56]  USA  Early Childhood Longitudinal Study - Birth Cohort | Cross-Sectional | N=8,950  Age: 4 years of age | **Nighttime sleep duration** was calculated from parental report. Two sleep groups were created as follows: those sleeping less than 1 standard deviation below the mean and all others. | Parents rated their child on 6 different externalizing behaviors (**overactivity, anger, aggression, impulsivity, tantrums,** and **annoying behaviors**) on a scale from 1 to 5 using the Preschool and Kindergarten Behavior Scale - Second Edition. | The adjusted odds ratios for children sleeping <9.44 hours (1 standard deviation below the mean) vs. those sleeping ≥9.44 hours are: overactivity = 1.30 (95% CI 1.03-1.65), anger = 1.40 (95% CI 1.15-1.71), aggression = 1.81 (95% CI 1.36-2.41), impulsivity = 1.44 (95% CI 1.12-1.86), tantrums = 1.46 (95% CI 1.16-1.85), and annoying behaviors = 1.40 (95% CI 0.97-1.87). | SES, parents in home, race, gender, TV viewing, and maternal depressive symptoms |
| Vaughn et al. [57]  USA | Cross-Sectional | N=62  Mean age: 4.15 y | **Sleep duration** was assessed using actigraphy (Ambulatory Monitoring Motionlogger) for at least 4 days. | A broad range of social/emotional measures including **emotion understanding, peer acceptance, social skills** and **social engagement** were assessed. Various tools were used to assess these dimensions including sociometric tasks, the California Child Q-sort, the Child Behavior Questionnaire, the Head to Toes task and a modified version of Denham’s emotion knowledge tasks. | Sleep duration was positively associated with peer acceptance, social skills, social engagement and understanding of the causes of emotion. | Sex, age, ethnicity |
| Liu et al. [64]  China | Cross-sectional | N = 513  Mean age: 4.46 y | **Sleep duration** was assessed using the Children’s Sleep Habits Questionnaire | The Strengths and Difficulties Questionnaire (SDQ) was used to assess psychopathology including **hyperactivity, peer problems, conduct problems, emotional symptoms** and **prosocial behavior** | Sleep duration was associated with hyperactivity (r=0.14, p<0.01), prosocial behavior (r=-0.17, p<0.01), conduct problems (r=0.14, p<0.01), emotional problems (r=0.22, p<0.01) and peer problems (r=0.20, p<0.01) | None |
| Yokomaku et al. [62]  Japan | Cross-Sectional | N=68 in Group A and N=67 in Group B  Mean age: 4.7 y (Group A) and 4.6 y (Group B)  Group A met one or more of the following conditions: they went out from their home with adults after 21:00 h two or more times a week, they went to bed after 23:00 h four or more times a week, and they returned home after 21:00 h three or more times a week, while those in Group B met none of these conditions. | Sleep-wake logs were completed daily for two weeks to assess **sleep duration**. | The Child Behavior Checklist was completed and assessed **withdrawn, somatic complaints, anxious/depressed, social problems, thought problems, attention problems, delinquent behavior,** and **aggressive behavior.** **Internalizing, externalizing**, and **total scale scores** were also derived. Generally, the higher the score, the greater the likelihood of problematic behaviors in that scale. | The Child Behavior Checklist score of the total scale was significantly higher in Group A than Group B (54.0 ± 9.1 vs. 49.8 ± 8.2, p<0.01). Children in Group A had significantly higher scores for withdrawn, anxious/depressed, aggressive behavior, internalizing and externalizing. However, total sleep duration did not correlate with the total Child Behavior Checklist score or with individual items. | None |
| ***Outcome: Cognitive Development*** | | | | | | |
| Giganti et al. [65]  Italy | Cross-Over Trial | N=23  Mean age: 52.6 months | This study tested the effect of a daytime nap on memory consolidation in preschoolers. After a study phase in which children had to name 40 pictures of objects and animals, each participant either took an actigraphically monitored nap or stayed awake. At retest, children were administered both an implicit and an explicit memory task. **Duration of daytime naps** were recorded at school by means of actigraphy, using Actiwatch-Plus actimeters. | Participants performed both a figures recognition task and a priming task in order to differentiate effects on explicit and implicit memory. The **implicit memory** task consisted of naming 40 pictures presented at 8 ascending levels of spatial filtering. The **explicit memory** task consisted of judging 40 pictures as old or new. | The number of correct answers at the explicit recognition task was significantly higher in the nap (87.4% hits) compared to the wake (sleep restriction) condition (77.4% hits) (t=2.37, df=22, p=0.02), whereas priming effects did not differ between conditions. | None |
| Bernier et al. [66]  Canada | Longitudinal | N=65  Mean age: T1: 12.9 months; T2: 26.3 months; T3: 48.9 months | The mother completed a 3-day sleep diary when infants were aged 1 year and **total daily sleep duration** was obtained. | Children completed two subscales of the Wechsler Preschool and Primary Scale of Intelligence at 4 years, indexing **general cognitive ability** and **complex executive functioning**. | Children getting higher proportions of their sleep at night as infants (i.e. 1 year), but not total sleep duration, were found to perform better on executive functions 3 years later, but did not show better general cognition. | Family SES and prior cognitive functioning |
| Bernier et al. [67]  Canada | Longitudinal | N=60  Age: T1: 12.9 months; T2: 18.3 months; T3: 26.3 months | A parent sleep diary was used when children were 12 and 18 months old. The sleep diary was completed by mothers on 3 consecutive days to estimate **total sleep duration.** | Child **executive functioning** was assessed at 18 months and 26 months to assess **working memory**, **inhibitory control**, and **set shifting** using various tasks. It was videotaped for later coding. Child overall **cognitive ability** was assessed at all time points (i.e. **mental development** at T1 and **expression vocabulary** at T2 and T3). At T1, children’s general cognitive functioning was assessed with the Mental Development Index of the Bayley Scales of Infant Development. At T2 and T3, expressive vocabulary was assessed using the MacArthur Communicative Development Inventory. | Higher proportions of total sleep occurring at night time, at both 12 and 18 months, were associated with better performance on executive tasks, especially those involving a strong impulse control component. However, the total sleep duration at 12 and 18 months was not associated with executive functioning at 18 and 26 months. Sleep duration at 12 months was not correlated with 18 month working memory (r=-0.11, p>0.05), 26 month conflict executive functioning (r=-0.10, p>0.05) or 26 month impulse control (r=-0.06, p>0.05). Sleep duration at 18 months was not correlated with 18 month working memory (r=-0.16, p>0.05), 26 month conflict executive functioning (r=0.09, p>0.05) or 26 month impulse control (r=-0.16, p>0.05). | Family SES, prior mental development and concurrent verbal ability |
| Horvath and Plunkett [68]  UK | Longitudinal | N=246  Mean age: 17.9 months  Follow-up: 3 and 6 months after the initial assessment | The Sleep and Naps Oxford Research Inventory, a sleep diary designed to be completed over 10 days, was completed by parents to assess **sleep duration**. | Vocabulary development was assessed using the Oxford Communicative Development Inventory. Comprehension (i.e. **receptive vocabulary**) and production (i.e. **expressive vocabulary**) scores are derived by counting the number of words the child is assumed to understand or say. | The length of nighttime sleep was negatively associated with rate of predicted expressive vocabulary growth (p=0.045). | Age, sex, breastfeeding |
| Jung et al. [69]  USA | Longitudinal | N=67  Age: 3 y, 4 y, and 5 y (mean ages: 42.1 months, 53.6 months, and 65.5 months) | The Sleep Questionnaire (parental report questions) was used to assess **sleep duration** at age 3. Sleep duration was dummy-coded as 6-7 h or less vs. 8-9 h or more. In addition, sleep duration was dummy-coded as less than 8-9 h vs. more than 10 h. | Scores on the General Conceptual Ability from the Differential Ability Scales were obtained at ages 3, 4, and 5 to assess the **development of cognitive skills**. | Children who had 8 h or more of sleep had significantly higher GCA scores than those with 7 h or less of sleep by 35.53 points at age 3. Children with more than 10 h of sleep had higher GCA scores at age 3 compared to children with 8-9 h or less of sleep (233.91 vs. 203.92, respectively). | None |
| Mindell and Lee [51]  Brazil | Cross-Sectional | N=1,351  Mean age: 7.4 months | The Brief Infant Sleep Questionnaire was filled out by the mothers and used to assess **sleep duration**. | The Ages and Stages Questionnaire was filled out by the mothers to assess **communication** and **problem solving skills.** | No significant associations were observed between nighttime sleep duration and communication and problem solving skills (p>0.05, coefficients not reported). | Child age, gender, and maternal education |
| Konrad et al. [70]  Germany | Cross-Sectional | N=48 (24 6-month-old and 24 12-month-old infants)  Mean age: 6-month-olds: 186 days; 12-month-olds: 365 days | **Sleep duration** was monitored for 24 h using actigraphy worn on the ankle (Micro Motionlogger Actiwatches, Ambulatory Monitoring Inc). Also, parents were asked to complete a sleep diary and **sleep duration** was calculated. | **Memory encoding** was assessed using an imitation task (puppet stimuli). Each session was video recorded and an imitation score was obtained. | Total sleep duration during the day and total sleep within 24 hours were not significantly associated to the adjusted imitation score at 6 and 12 months (correlation coefficients for both groups: r=0.25, p=0.245). For 6 months age group: r=0.14, p>0.05. For 12 months age group: r=0.09, p>0.05. | Co-sleeping status |
| Scher [71]  Israel | Cross-Sectional | N=50  Age: 10-month-olds | **Sleep duration** was assessed using both actigraphy worn on the ankle (miniature actigraphs, Ambulatory Monitoring) over 3 consecutive days and using a sleep questionnaire. | The Bayley Scales of Infant Development was used to assess both the **mental development index** (MDI) and the **psychomotor development index** (PDI). | Sleep duration was not associated with MDI (r=-0.09, p>0.05) or PDI (r=0.08, p>0.05). | None |
| Gibson et al. [38]  New Zealand | Cross-Sectional | N=52  Age: 11-13 months | Infant **sleep duration** was assessed by one week’s actigraphy worn on the calf (AW64 actiwatch, Respironics and Mini Mitter Inc), by sleep diaries and by the Brief Infant Sleep Questionnaire. | The Ages and Stages Questionnaire was completed by parents to assess **communication and problem solving skills.** | Sleep duration was not associated with communication and problem solving skills (non-significant results were not reported). | Age and gender. |
| Lukowski and Milojevich [72]  USA | Cross-Sectional | N=25  Mean age: 316 days | Parents completed the Brief Infant Sleep Questionnaire to assess **sleep duration**. | Infants were presented with six 2-step event sequences to assess **recall memory** and **generalization**. **Elicited imitation** testing was video recorded and coded by an experienced coder. | Nighttime sleep duration was not associated with elicited imitation scores. Correlation between night time sleep duration with baseline target action (r=-0.30); with baseline pairs of actions (r=0.11); with immediate imitation of target actions (r=0.28); with immediate imitation of pairs of actions (r=-0.08); with delayed recall of target actions (r=0.19); and with delayed recall of pairs of actions (r=0.21). p>0.05 for all correlations. | Baseline performance |
| Hoyniak et al. [73]  USA | Cross-Sectional | N=15  Mean age: 2.79 y | **Sleep duration** was assessed for the week preceding testing using Actigraphs worn primarily on the wrist (no brand provided). | **Attentional processing** was assessed using an auditory Oddball task while EEG data were collected. | There was no significant association between sleep duration and the outcome variables (neural processing and sustained attention) (all p>0.05) | Number of target trials, percent of correct target trials and age |
| Keefe-Cooperman and Brady-Amoon [55]  USA | Cross-Sectional | N=874  Mean age: 40.0 months | Parents or caregivers were asked to indicate their child’s **total hours of nighttime sleep** as part of a structured interview protocol. | The Stanford-Binet 5 instrument was used to assess **cognitive functioning**. The three variables investigated were the full scale IQ scores, verbal IQ scores and nonverbal IQ scores. | Total nighttime sleep was not associated with cognitive functioning: norm reference FSIQ score (r=0.03, p>0.05), composite verbal score (r=0.05, p>0.05) and composite nonverbal score (r=0.03, p>0.05). | None |
| Vaughn et al. [57]  USA | Cross-Sectional | N=62  Mean age: 4.15 y | **Sleep duration** was assessed using actigraphy (Ambulatory Monitoring Motionlogger) for at least 4 days. | **Receptive vocabulary** (PPVT-IV) and **attention focus** (Child Behavior Questionnaire). | Sleep duration was significantly associated with receptive vocabulary (r=0.45, p<0.01) but not with attention focus. | Sex, age, ethnicity |
| Lam et al. [74]  USA | Cross-Sectional | N=59  Mean age: 4.3 y | Participants wore an actigraph watch for 7 days to assess **sleep duration** (MicroMini Motion-logger Actigraph watch). | **Cognitive function** was evaluated using neuropsychological tests including **attention**, **response control**, and **vocabulary**. Tests included the Peabody Picture Vocabulary Test, Fourth Edition (PPVT-IV), the Number Recall (Kaufman Assessment Battery for Children, Second Edition), the Statue test, and the Auditory Continuous Performance Test for Preschoolers (ACPT-P). | Sleep duration was positively correlated with vocabulary in the PPVT-IV test (r=0.29, p=0.03), such that more nighttime sleep was associated with better vocabulary. Sleep duration was negatively correlated with ACPT-P errors of commission (r=-0.29, p=0.03), such that those who slept less at night made more impulsive errors on a computerized go/no-go test. However, no associations were found between sleep duration and the Number Recall (r=-0.05) or the Statue Test (r=0.13). | Age |
| Nathanson et al. [76]  USA | Cross-Sectional | N=107  Mean age: 53.4 months | Parents completed a questionnaire assessing **sleep duration** of their child. | **Executive function** was assessed via one-on-one interviews using four tasks: grass/snow task, whisper task, backward digit span task, and tower task. | Sleep duration was not associated with executive function at the zero-order level. In the most adjusted model (model 3 in the hierarchical regression analysis), sleep duration was found negatively associated with executive function: β=-0.28, SE=0.24, p<0.01. | None |
| Scott et al. [75]  England  Avon Longitudinal Study of Parents and Children | Cross-Sectional | N=8,195  Age: 6, 18, 42, 69, 81, 115 and 140 months | Parental questionnaire asked questions about sleep patterns (including **total sleep duration**) at all time points. | The Development and Well-Being Assessment was used to assess attention deficit hyperactivity disorder (**ADHD**). This assessment is a package of interviews, questionnaires and rating techniques. The questionnaires of children with symptoms of ADHD were then rated by an experienced child psychiatrist to confirm DSM-IV diagnoses. | At every evaluation, children with ADHD and related subtypes had lower sleep duration compared with the rest of the cohort, and this was more marked during the younger primary school years. Using a multivariable linear regression model and adjusting for covariates, total sleep duration was significantly less for ADHD children at 69 months (by 12 min), 81 months (by 15 min) and 115 months (by 11 min). | Gender, maternal age, pre-term births and larger families |
| ***Outcome: Motor Development*** | | | | | | |
| Mindell and Lee [51]  Brazil | Cross-Sectional | N=1,351  Mean age: 7.4 months | The Brief Infant Sleep Questionnaire was filled out by the mothers and used to assess **sleep duration**. | The Ages and Stages Questionnaire was filled out by the mothers to assess **gross and fine motor skills**. | No significant associations between nighttime sleep duration and gross and fine motor skills were seen (p>0.05, coefficients not reported). | Child age, gender, and maternal education |
| Gibson et al. [38]  New Zealand | Cross-Sectional | N=52  Age: 11-13 months | Infant **sleep duration** was assessed by one week’s actigraphy worn on the calf (AW64 actiwatch, Respironics and Mini Mitter Inc), by sleep diaries and by the Brief Infant Sleep Questionnaire. | The Ages and Stages Questionnaire was completed by parents to assess **gross and fine motor skills.** | Sleep duration was not associated with gross and fine motor skills (non-significant results were not reported). | Age and gender. |
| ***Outcome: Growth*** | | | | | | |
| Lampl et al. [29]  USA | Longitudinal | N=23  Age: 4-17 months | Daily parental diaries continuously recorded **sleep duration** over 4 to 17 months. | Growth in **total body length** was assessed using the maximum stretch technique. | Saltatory length growth was associated with increased total daily sleep hours (p<0.001) and number of sleep bouts (p=0.001). Subject-specific probabilities of a growth saltation associated with sleep included a mean odds ratio of 1.20 for each additional hour (n=8, 95% CI 1.15-1.29) and 1.43 for each additional sleep bout (n=12, 95% CI 1.21-2.03). | Breastfeeding, sex and age |
| Tikotzky et al. [77]  Israel | Cross-Sectional | N=96  Age: 6-month-old infants | Infant **sleep duration** was assessed by actigraphy (Ambulatory Monitoring Inc) attached to the ankle for 4 consecutive nights and by the Brief Infant Sleep Questionnaire. | Infant’s weight and length measures were assessed during a standard checkup at the infant-care clinic when the infants were 6 months old. **Weight-to-length ratio** (WLR) was calculated and **weight above expected weight for length** (WEFL) was obtained. | Using actigraphy, sleep duration was associated with WLR (r=-0.47, p<0.01) in girls only. Using the questionnaire, night sleep duration was associated with WLR (r=-0.26, p<0.05) and WEFL (r=-0.25, p<0.05) in the total sample. | Gestational age, birth weight, breast feeding, sleep position, parental education, parental age and number of rooms at home (as an indicator of SES) |
| ***Outcome: Sedentary Behavior*** | | | | | | |
| Magee et al. [22]  Australia  Longitudinal Study of Australian Children | Longitudinal | N=2,984  Age: Children were followed from 4 to 5 years of age until 8 to 9 years of age | Time-use diaries were used to assess **sleep duration**. At each 3 waves, the parent completed two diaries; one encompassed a 24-h period on a weekday, the other a 24-h period on a weekend day. | **Television viewing** (watching television, video, DVD, movie) and **computer usage** (using a computer/computer games) were derived from time-use diaries. At each 3 waves, the parent completed two diaries; one encompassed a 24-h period on a weekday, the other a 24-h period on a weekend day. | Sleep duration at 4 years of age was inversely associated with television viewing (β=-0.07, p=0.003) and computer use (β=-0.04, p=0.001) at 6 years of age. | Sex, sleep problems, household income, maternal education, maternal weight status |
| Plancoulaine et al. [31]  France  EDEN Study | Cross-Sectional | N=1,028  Age: 3 years of age | A parental self-administered questionnaire was used to assess **sleep duration**. Short sleepers were defined as children with total sleep duration of less than 12 h per day. | The number of **hours per day spent watching television or other screens** during a usual week were collected by the parents separately for weekdays, Wednesdays (weekday without school in France), and for weekend days. | Short sleep duration was associated with time spent watching TV (OR: 1.65, 95% CI 1.23-2.21 per additional hour/24 h) in boys. In girls, the association was not significant (p=0.75). | Centre, familial income, educational level, child-care arrangement, isolated mother, maternal BMI, maternal depression, night awakenings, parental presence when falling asleep, food patterns, BMI z scores |
| Vijakkhana et al. [78]  Thailand | Cross-Sectional | N=208  Age: 6.28 months and 12.30 months | A sleep diary was used to assess the infant’s sleep onset and wake time at 6 and 12 months of age (completed by a primary caregiver). **Nighttime sleep duration** was then calculated separately for weekdays and weekend days at both ages. | **Screen media exposure** in the household was assessed at 6 and 12 months of age using a media diary completed by the parents and caregivers. | Infants who were exposed to screen media in the evening at 12 months of age had a 28-min lower nighttime sleep duration on weekdays. Moreover, infants who were exposed to screen media in the evening at age 6 months and 12 months had shorter 12-month nighttime sleep duration compared with those who were not exposed to screen media after 7pm at both ages. | 12-month estimated media viewing, chronological age, gender, co-sleeping status, maternal education in years, and income |
| McDonald et al. [79]  UK  Gemini Study | Cross-Sectional | N=1,702  Mean age: 15.8 months | Sleep duration was assessed with a modified version of the Brief Infant Sleep Questionnaire by the primary caregiver. **Nighttime sleep duration** was calculated from bedtime and wake time, and short sleep duration was defined as less than 11 hours per night. | **TV viewing** was reported by the primary caregiver in a baseline questionnaire. Hours of TV viewing in the morning and after 6:30 in the evening were obtained and categorized as ≤1 and >1 hour. | Watching more than an hour of TV in the evening was associated with short sleep duration (OR=1.89, 95% CI 1.26-2.84). However, the association was not significant with watching more than an hour of TV in the morning (OR=1.13, 95% CI 0.80-1.58). | Age, sex, maternal education, ethnicity, birth weight, number of older children, daytime sleep, and regular night waking |
| Ikeda et al. [80]  Japan  Longitudinal Survey of Babies in the 21st Century and Live Birth Forms of the Vital Statistics Survey | Cross-Sectional | N=39,813  Mean age: 4.5 years | **Sleep duration** was assessed as part of a questionnaire filled out by the parents. Short sleepers were defined as children who slept less than 10 hours per day. | **Hours spent watching TV and playing computer games** was reported by the parents in a questionnaire. | Short sleep duration was associated with longer hours spent watching television (OR=1.91, 95% CI 1.26-2.90 for ≥4 h/day) and playing computer games (OR=1.62, 95% CI 1.18-2.23 for ≥2 h/day) compared to not watching/playing. | Regional population, gender, existence of older siblings, years of maternal and paternal education, paternal and maternal work hours, and whether or not the child attended preschool or a childcare center |
| ***Outcome: Physical Activity*** | | | | | | |
| Magee et al. [22]  Australia  Longitudinal Study of Australian Children | Longitudinal | N=2,984  Age: Children were followed from 4 to 5 years of age until 8 to 9 years of age | Time-use diaries were used to assess **sleep duration**. At each 3 waves, the parent completed two diaries; one encompassed a 24-h period on a weekday, the other a 24-h period on a weekend day. | **Physical activity** (time spent engaged in organized sport/physical activity or active play) was derived from time-use diaries. At each 3 waves, the parent completed two diaries; one encompassed a 24-h period on a weekday, the other a 24-h period on a weekend day. | Sleep duration at 4 years of age was not associated with physical activity at 6 years of age (β=-0.02, 95% CI -0.09-0.03). | Sex, sleep problems, household income, maternal education, maternal weight status |
| Hager et al. [30]  USA | Cross-Sectional | N=240  Mean age: 20.2 months | Mothers completed the Brief Infant Sleep Questionnaire to assess **nighttime sleep duration.** | **Physical activity** was objectively assessed using an Actical accelerometer on the nondominant or left ankle for 7 consecutive days. | Longer nighttime sleep duration was associated with more physical activity (MVPA min/day: r=0.19, p=0.012; activity counts: r=0.21, p=0.006). In multivariable models, nighttime sleep duration was positively associated with physical activity (β=0.332, p=0.017). | Age, sex, maternal marital status, and recruitment location |
| Plancoulaine et al. [31]  France  EDEN Study | Cross-Sectional | N=1,028  Age: 3 years of age | A parental self-administered questionnaire was used to assess **sleep duration**. Short sleepers were defined as children with total sleep duration of less than 12 h per day. | The number of **hours per day spent in physical activity** (walking, playing outside) during a usual week was collected by the parents separately for weekdays, Wednesdays (weekday without school in France), and for weekend days. | Sleep duration was not associated with physical activity in either boys (p=0.89) or girls (p=0.41). | Centre, familial income, educational level, child-care arrangement, isolated mother, maternal BMI, maternal depression, night awakenings, parental presence when falling asleep, food patterns, BMI z scores |
| Hinkley et al. [81]  Australia  Healthy Active Preschool Years Study | Cross-Sectional | N=1,004  Mean age: 4.5 y | Parents completed a comprehensive survey, including a question on **sleep duration**. | **Physical activity level** was measured using accelerometers (ActiGraph GT1M) over 8 days. | Total daily sleep duration was positively associated with physical activity in boys only (OR=1.04, 95% CI 1.02-1.07). | Clustering by center of recruitment |
| ***Outcome: Quality of Life/Well-Being*** | | | | | | |
| Wang et al. [82]  Japan  Toyama Birth Cohort Study | Longitudinal | N=9,674  Age: 3 years of age followed until first-year junior high school (approx. 13 years old) | **Sleep duration** was assessed in a questionnaire survey sent to the parents. Sleep duration was categorized as less than 10 h, 10-11 h, and 11 h or more. | Quality of life was assessed using COOP charts and consisting of 9 single-item subscales covering separate dimensions of health-related quality of life (physical fitness, daily activities, social activities, pain, change in health, social support, feelings, overall health, and quality of life). The chart “**Quality of life**” was used in this study (five response categories from very well to very bad). | Short sleep duration at 3 years of age (<10 h vs. >11 h) was not associated with poor quality of life in first-year junior high school (OR=1.15, 95% CI 0.99-1.33, p=0.06). | Age, gender, BMI and family factors at 3 years of age |
| ***Outcome: Risks/Injuries*** | | | | | | |
| Koulouglioti et al. [83]  USA  Rochester Preschool Children Injuries study | Cross-Sectional | N=278  Age: 18 months to 4 years of age | Mothers reported **sleep duration** as part of a questionnaire (including naps) and also completed the 3-item subscale on sleep duration from the Children’s Sleep habits Questionnaire. | The main outcome measure was the **total number of medically attended injuries** between 18 months and 4 years of age. This included all injuries for which either self-report or medical record data were available at either measurement point. | Children with shorter sleep duration sustained a higher number of medically attended injuries (b=0.1759, p<0.05). | Mother’s education, mother’s age, and child’s temperament |
| Boto et al. [84]  Portugal | Cross-Sectional | N=2,033  Age: 1-14 years (6.1 y in G1 and 4.9 y in G2)  G1: children observed in an emergency room for risk of accidental falls.  G2: children attending health care visits. | In both groups, parents or caregivers answered a questionnaire. The child’s **average sleep duration** in the previous week was assessed through a closed question with three levels: less than 8 h per day, 8-10 h per day, or more than 10 h per day (considering 8 h per day as insufficient sleep duration and 10 h as the desirable sleep period). | **Risk of accidental falls** was the outcome variable. In G1 the interview was conducted by doctors and nurses on call in the pediatric emergency room. In G2 family nurses at the ambulatory clinic were responsible for its completion. Several questions were asked to characterize the accident, including mechanism of fall and injury severity. | Usual sleep duration shorter than 8 hours was associated with an increased risk of accidental falls (OR=2.7, 95% CI 1.2-6.1). | Gender, age, paternal education level, mother’s professional level, summer holidays, lack of daytime nap, and sleeping less than usual |
| Owens et al. [85]  USA | Cross-Sectional | N=71  Mean age: 4.74 years | **Sleep duration** was assessed using the Children’s Sleep Habits Questionnaire. | **Injury risk** was assessed using the Injury Behavior Checklist and chart review of injuries. The high-injury-history group was defined as ≥2 injuries over the previous 24 months and the low-injury-history group was defined as 0 to 1 injury over the previous 24 months. In addition, the high-injury behavior (Injury Behavior Checklist total score >1 SD above the group mean score) and low-injury behavior (Injury Behavior Checklist score total score ≤1 SD above the group mean score) were used. | The CSHQ sleep duration score did not significantly differ between the high injury and low injury groups (5.93 ± 1.03 vs. 6.36 ± 0.96, respectively, p=0.09). Also, the CSHQ sleep duration score did not significantly differ between the high-injury-behavior and the low-injury-behavior groups (5.73 ± 2.10 vs. 4.32 ± 1.92, respectively, p not provided) after Bonferroni correction. The Pearson correlation coefficient between sleep duration and the total Injury Behavior Checklist score was r=0.32, p=0.005. To specifically examine the relationship between parent-reported sleep duration and injuries and injury behavior, they divided the group by median split for sleep duration (low sleep <690 min, high sleep ≥690 min). There were no significant differences in the number of injuries in the past 2 years or in the Injury Behavior Checklist total score. | Age |

**Abbreviations:**

ADHD, attention deficit hyperactivity disorder; ACPT-P, Auditory Continuous Performance Test for Preschoolers; BASC, Behavior Assessment System for Children; BMI, body mass index; CAR, cortisol awakening response; CDC, Centers for Disease Control and Prevention; CI, confidence interval; DEXA, dual-energy X-ray absorptiometry; ECBQ, Early Childhood Behavior Questionnaire; EEG, electroencephalography; FFM, fat-free mass; FM, fat mass; GCA, General Conceptual Ability; IOTF, International Obesity Task Force; IQ, intellectual quotient; MDI, mental development index; OB, obese; OR, odds ratio; OW, overweight; PDI, psychomotor development index; PPVT, Peabody Picture Vocabulary Test; PR, prevalence ratio; PSG, polysomnography; SDQ, Strengths and Difficulties Questionnaire; SES, socio-economic status; WEFL, weight above expected weight for length; WLR, weight-to-length ratio; WHO: World Health Organization.
